# Rac1-Dependent Collective Cell Migration Is Required for Specification of the Anterior-Posterior Body Axis of the Mouse

**DOI:** 10.1371/journal.pbio.1000442

**Published:** 2010-08-03

**Authors:** Isabelle Migeotte, Tatiana Omelchenko, Alan Hall, Kathryn V. Anderson

**Affiliations:** 1 Developmental Biology Program, Sloan-Kettering Institute, New York, New York, United States of America; 2 Cell Biology Program, Sloan-Kettering Institute, New York, New York, United States of America; Duke University Medical Center, United States of America

## Abstract

Cell migration and cell rearrangements are critical for establishment of the body plan of vertebrate embryos. The first step in organization of the body plan of the mouse embryo, specification of the anterior-posterior body axis, depends on migration of the anterior visceral endoderm from the distal tip of the embryo to a more proximal region overlying the future head. The anterior visceral endoderm (AVE) is a cluster of extra-embryonic cells that secretes inhibitors of the Wnt and Nodal pathways to inhibit posterior development. Because Rac proteins are crucial regulators of cell migration and mouse *Rac1* mutants die early in development, we tested whether Rac1 plays a role in AVE migration. Here we show that *Rac1* mutant embryos fail to specify an anterior-posterior axis and, instead, express posterior markers in a ring around the embryonic circumference. Cells that express the molecular markers of the AVE are properly specified in *Rac1* mutants but remain at the distal tip of the embryo at the time when migration should take place. Using tissue specific deletions, we show that Rac1 acts autonomously within the visceral endoderm to promote cell migration. High-resolution imaging shows that the leading wild-type AVE cells extend long lamellar protrusions that span several cell diameters and are polarized in the direction of cell movement. These projections are tipped by filopodia-like structures that appear to sample the environment. Wild-type AVE cells display hallmarks of collective cell migration: they retain tight and adherens junctions as they migrate and exchange neighbors within the plane of the visceral endoderm epithelium. Analysis of mutant embryos shows that Rac1 is not required for intercellular signaling, survival, proliferation, or adhesion in the visceral endoderm but is necessary for the ability of visceral endoderm cells to extend projections, change shape, and exchange neighbors. The data show that Rac1-mediated epithelial migration of the AVE is a crucial step in the establishment of the mammalian body plan and suggest that Rac1 is essential for collective migration in mammalian tissues.

## Abstract 2

Live imaging and analysis of conditional mutants show that the embryonic organizer that determines the anterior-posterior axis in the mouse embryo moves by Rac1-dependent collective cell migration.

## Author Summary

The specification of the anterior-posterior body axis of the mouse embryo depends on migration of the anterior visceral endoderm (AVE) to a position that overlies the future head. By high-resolution imaging of intact embryos we show that movement of the AVE is a form of collective cell migration, as the migrating cells retain tight and adherens junctions while they migrate and exchange neighbors within the plane of the visceral endoderm epithelium. Using conditional knockouts, we find that the small GTPase Rac1 is absolutely required for specification of the anterior-posterior axis and acts cell-autonomously within the AVE to allow cells to extend long, dynamic lamellar projections that are required for movement. Rac1-mediated epithelial migration of the AVE is a crucial step in the establishment of the mammalian body plan, and Rac1 may be important for collective migration in general in mammalian tissues, including invading tumor cells.

## Introduction

Between the time of implantation and gastrulation, the pluripotent cells of the mammalian epiblast become restricted to specific lineages in a series of inductive interactions that depend on both intercellular signals and highly orchestrated cell rearrangements. One day after implantation (e5.5), the embryonic region that will give rise to the three germ layers of the mouse is a single-layered cup-shaped columnar epithelium (the epiblast) that is surrounded by the squamous visceral endoderm (VE) epithelium. At this stage, the mouse embryo is elongated in its proximal-distal axis, where the site of connection to the uterine tissue defines the proximal pole. Proximal-distal differences in the pattern of gene expression first become apparent at e5.5, when a group of VE cells at the distal tip of the embryo (the distal visceral endoderm (DVE)) expresses a distinctive set of molecular markers, including the transcription factor Hex. Between e5.5 and e6.0, this population of cells migrates proximally and comes to lie on the presumptive anterior side of the embryo, adjacent to the embryonic/extra-embryonic boundary [1],[2], where the cells are known as the anterior visceral endoderm (AVE). The cells of the AVE secrete localized Nodal and Wnt inhibitors that confine Wnt and Nodal signals to the opposite side of the embryo, where the primitive streak is then specified. Thus migration of DVE/AVE cells converts the early proximal-distal asymmetry into the definitive anterior-posterior (AP) axis of the animal.

Although migration of mammalian cells has been studied extensively in culture, little is known about the dynamics of cell migration in intact mouse embryos. As the AVE lies on the surface of the embryo and AVE migration is completed in about 5 h, it has been possible to image migration of AVE cells in vivo [2]. The AVE is therefore ideal for studies of the cell behaviors and the genetic control of mammalian cell migration in vivo.

The mechanisms of AVE migration are the subject of considerable debate. It has been observed that migrating AVE cells extend filopodia in the direction of movement, which suggests that they may migrate towards an unidentified chemoattractant [2] or away from a chemorepellant [3]. Alternatively, it has been proposed that the cells might migrate in response to instructive cues from the extracellular matrix [4],[5]. The non-canonical Wnt pathway proteins Celsr1 and Testin are expressed in the AVE [6], which suggests that AVE cells might move through planar polarity-dependent cellular rearrangements analogous to those that take place in the extending germ band of *Drosophila* [7],[8]. In mouse embryos that lack Prickle1, a non-canonical Wnt pathway protein, the AVE fails to move; however, this defect is accompanied by a disruption of apical-basal polarity, so it is not clear whether Prickle1 regulates AVE migration directly [9].

We showed previously that the actin regulator Nap1, a component of the WAVE complex, is important for AVE migration [10]. The WAVE complex is crucial for migration of many cell types; it promotes the formation of branched actin networks at the leading edge of migrating cells and thereby pushes the cell membrane forward [11]–[13]. The ENU-induced *Nap1^khlo^* allele was identified in a genetic screen for mutations that affect embryonic patterning [10]. The most dramatic phenotype of *Nap1^khlo^* mutants is the duplication of the AP body axis, seen in about 25% of the mutant embryos, and correlated with partial migration of the AVE. These studies indicated that WAVE-mediated migration was important for AVE migration and axis specification, but the low penetrance of the axis duplication phenotype left open the possibility that other, WAVE-independent mechanisms might be crucial for AVE migration. The WAVE complex can act downstream of Nck or Rac [14]; activation by Rac requires simultaneous interaction with acidic phospholipids [15]. Because the AP body axis is specified normally in mouse mutants that lack Nck [16], we hypothesized that the small GTPase Rac1 might act upstream of the WAVE complex to direct AVE migration.

The functions of mammalian Rac1 have been studied in a variety of processes. Conditional inactivation of *Rac1* in mouse fibroblasts has confirmed that Rac plays roles in lamellipodia formation, cell-matrix adhesion, and cell survival [17]. Tissue-specific gene inactivation experiments have also implicated mouse Rac1 in a variety of processes, including EGF-induced cell proliferation of the neural crest [18], canonical Wnt signaling during limb outgrowth [19], actin rearrangements required for myoblast fusion [20], regulation of p38 MAP kinase activity and of Indian Hedgehog expression in chondrocytes [21], and maintenance of stem cells in the skin through regulation of c-Myc expression [22]. Nevertheless, no genetic studies have addressed the roles of Rac proteins in vertebrate morphogenesis.

Although the mouse genome encodes three forms of Rac, only *Rac1* is expressed in the early embryo [23],[24]. Because the tissue organization of the early mouse embryo is relatively simple, we set out to define precise functions of Rac in tissue migration in vivo in the intact early embryo. It was previously shown that *Rac1* null embryos die at the time of gastrulation [25]. Here we show that *Rac1* is essential for the establishment of the AP axis of the mouse embryo. We use high-resolution imaging to show that wild-type AVE cells move in a coordinated fashion and extend long projections that can span nearly the entire embryonic region. In embryos that lack *Rac1*, AVE cells lack all projections and fail to move from their original location at the distal tip of the embryo. These findings demonstrate that Rac1 is a critical link that connects morphogenetic signals to AVE migration, allowing the establishment of the AP axis of the mammalian body plan.

## Results

### Rac1 Null Embryos Fail to Specify the AP Body Axis

It was previously described that *Rac1* null mutant embryos are delayed in growth as early as e5.75 and arrest between e6.0 and e7.5 [25]. We generated a null allele of *Rac1* by germ line deletion of a conditional allele [26]. Most (∼60%) of the *Rac1* null embryos arrested before e7.5, and all had arrested by e7.5; thus, this allele recapitulated the early lethality previously seen in *Rac1* null embryos.

**Figure 1:**
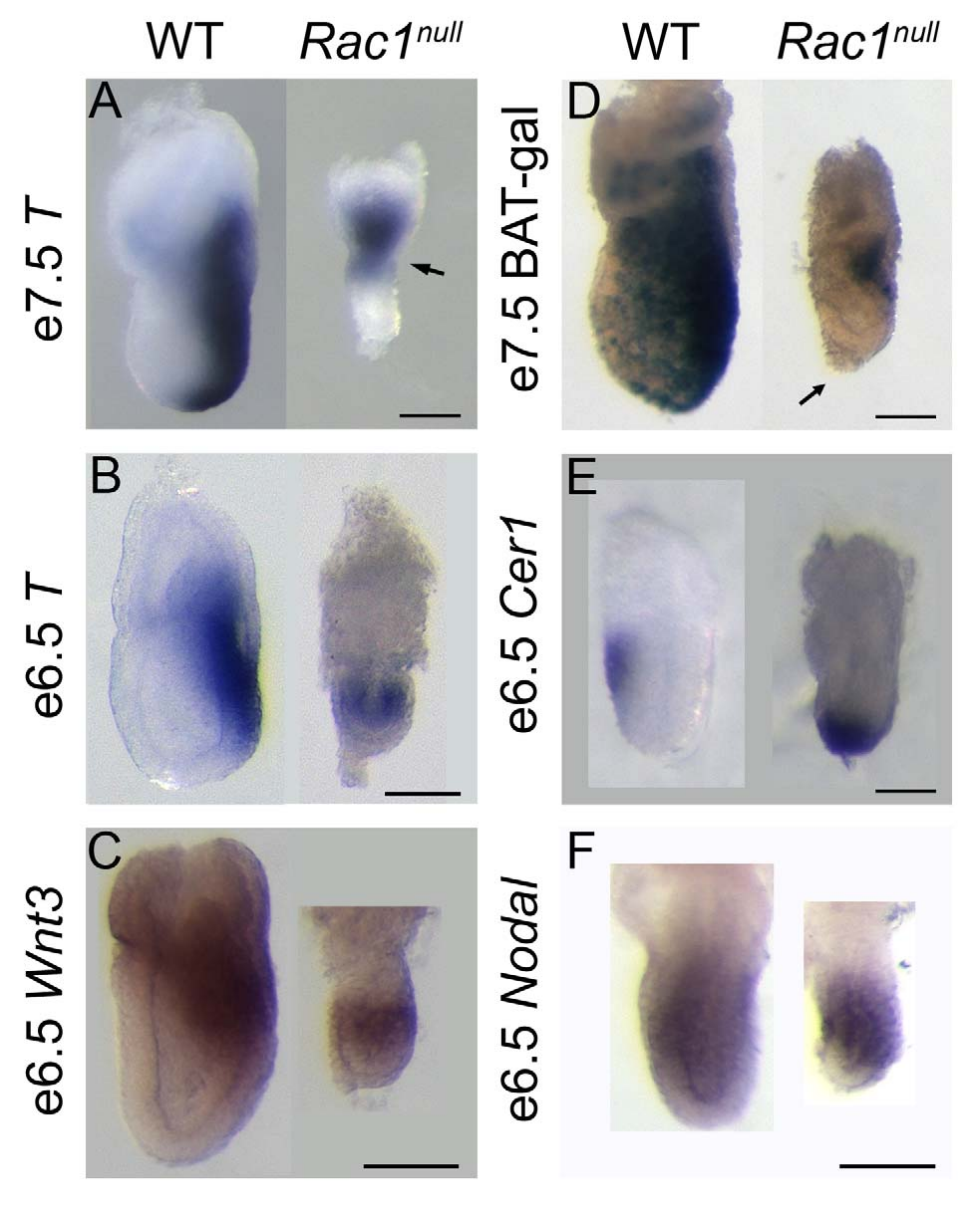
*Rac1 null* embryos fail to specify an AP axis. Expression markers of the primitive streak and AVE. (A) Wild-type e7.5 embryos expressed *Brachyury (T)* in the primitive streak, on the posterior side of the embryo. E7.5 *Rac1 null* embryos displayed a constriction at the embryonic/extra-embryonic boundary (arrow). They expressed *T* in a ring at the embryonic/extra-embryonic border (6/8) or only in the extra-embryonic region (not shown; 2/8). In 2 out of 8 embryos, *T* expression was slightly polarized to one side. (B) At e6.5, *T* was expressed in a spot (2/17) or ring (9/17) at the embryonic/extra-embryonic border of *Rac1 null* embryos. No expression was detected in 6/17 mutants, presumably due to developmental delay. (C) *Wnt3* was expressed around the circumference of *Rac1* null embryos at e6.5 (7/17; no staining in 10/17). (D) In 2/2 mutant embryos, the Wnt reporter BAT-gal was expressed in a slightly polarized fashion at the embryonic/extra-embryonic junction at e7.5. Note that in those embryos where the primitive streak was asymmetric, a cluster of AVE cells that failed to migrate (arrow) was slightly displaced from the embryo tip opposite to the position of streak marker expression, revealing a slight residual asymmetry. (E) *Cer1*, marking the AVE, is expressed distally at e6.5 in *Rac1 null* embryos. (F) *Nodal*, which is required for specification of the AVE, was expressed at e6.5 in a proximal-to-distal gradient in *Rac1 null* embryos. WT, wild-type. Scale bars = 100 µm in A, B, C, D, and F, and 50 µm in E. Anterior is to the left in all panels.

The *Rac1* null embryos that survived to e7.5 often showed a constriction at the boundary between embryonic and extra-embryonic regions (Figure 1A, arrow). This phenotype has been observed in mutants in which specification of the AP body axis is disrupted (e.g. *FoxA2* [27]; *Axin* [28]; *Nodal* [29]; *Otx2* [30]; *Lim1* [31]). We therefore examined expression of markers of early AP patterning in *Rac1* null embryos.

*Brachyury* (*T*), an early marker of the primitive streak, is expressed exclusively on the posterior side of wild-type e6.5–e7.5 embryos. *T* was expressed ectopically in e7.5 *Rac1* null embryos in a ring at the embryonic/extra-embryonic border (Figure 1A). One day earlier, at the onset of gastrulation (Figure 1B; e6.5), *T* was expressed in a spot or ring at the embryonic/extra-embryonic border in the mutants. *Wnt3* is the earliest marker of the primitive streak [32]. At e6.5, *Wnt3* was expressed on the posterior side of wild-type embryos and in a ring at the level of the embryonic/extra-embryonic boundary of *Rac1* mutant embryos (Figure 1C). Nodal, which is required for primitive streak formation, was expressed in e6.5 *Rac1* mutants (Figure 1F). Despite the ectopic expression of *T* and *Wnt3*, gastrulation did take place in the mutants, as shown by the formation of a mesodermal layer (Figure S1; [25]). In a minority of e7.5 *Rac1* embryos (∼30%), streak markers, such as *T* or the Wnt reporter BAT-gal (Figure 1D), did not encircle the circumference of the embryo and instead were restricted to one side of the embryo. The primitive streak never extended distally but remained as a spot at the embryonic/extra-embryonic boundary. Thus the processes that trigger primitive streak formation are ectopically initiated at all positions around the circumference of most *Rac1* null embryos.

### Axis Specification Fails Because AVE Cells Do Not Migrate in Rac1 Null Embryos

The position of the primitive streak depends on the earlier migration of the AVE cells from the distal tip of the embryo to the embryonic/extra-embryonic boundary. We therefore examined the expression of *Cer1*, a marker of the AVE. In wild-type embryos, *Cer1* expressing cells had moved from the distal tip to the embryonic/extra-embryonic boundary at e6.5. In contrast, in *Rac1* null embryos, *Cer1* expression was restricted to the distal tip (Figure 1E).

**Figure 2:**
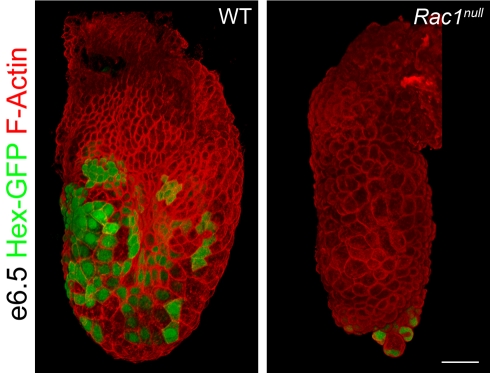
Hex-expressing AVE cells fail to migrate in *Rac1 null* embryos. 3D reconstruction of whole-mount confocal Z-stacks of e6.5 embryos stained with phalloidin to visualize F-actin (red). Expression of the Hex-GFP transgene (green) marks AVE cells at the end of migration. Hex-expressing cells reside at the embryonic/extra-embryonic boundary of wild-type embryos at this stage. In contrast, Hex-expressing cells are found in a cluster at the distal tip of the *Rac1* mutant. Hex-GFP was detected with anti-GFP antibody in wild-type and native GFP in the mutant. Scale bar = 50 µm.

Migration of AVE cells can be followed at cellular resolution using the *Hex-GFP* transgene, which is expressed specifically in the AVE when the cells are at the distal tip of the embryo and continues to be expressed during their migration [2]. Hex-GFP expression in *Rac1* null embryos was indistinguishable from that in wild-type embryos at e5.5, before AVE migration (not shown). While Hex-GFP-expressing cells in wild-type embryos had completed migration to the extra-embryonic border at e6.5, Hex-GFP-positive cells remained in their original position at the distal tip of the embryo in *Rac1* null embryos (Figure 2). The Hex-GFP expressing cells at the distal tip of the *Rac1* null embryos gradually formed a grape-like cluster (Figure 2, Figure S2B), as has been seen in other mutants in which AVE cells fail to migrate (*FoxA2* (Figure S2C, [27]), *Otx2* [30], *Nap1* (Figure S2D), and β-catenin [33]). Thus migration of the AVE cells fails in the absence of Rac1; the failure of AVE migration is sufficient to explain the failure to specify the primitive streak at a single position during gastrulation.

### Rac1 Acts in the VE to Promote AVE Migration

**Figure 3:**
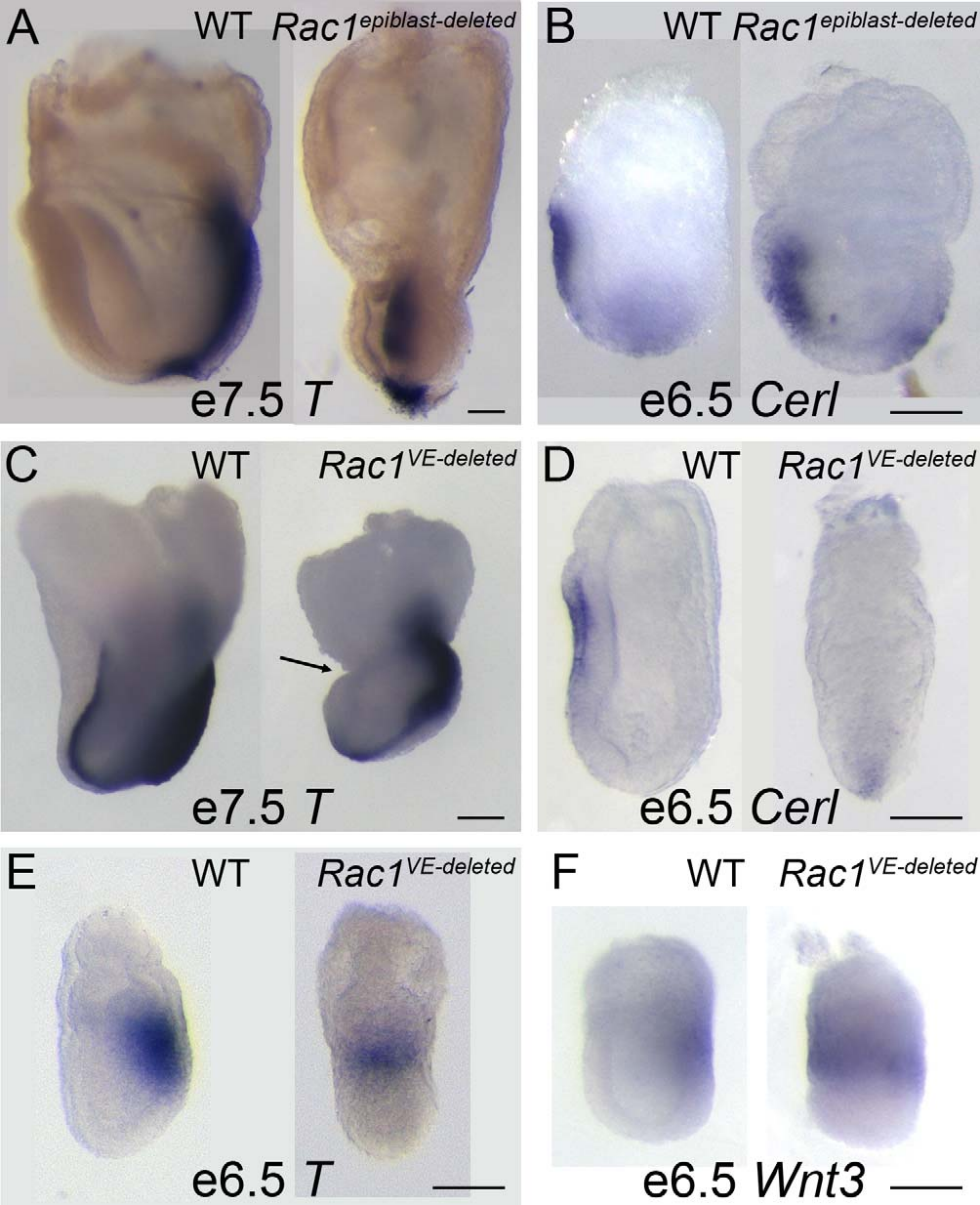
Rac1 acts autonomously to promote AVE migration. In situ hybridization with markers of the AVE and the primitive streak; lateral views of embryos are shown. (A) *T* was expressed normally in e7.5 *Rac1 epiblast-deleted* embryos, although the shape of the primitive streak was disrupted in the mutants. The *T*-positive primitive streak formed a bulge that filled most of the amniotic cavity, due to disruption of the behavior of the embryonic mesoderm ([25]; Migeotte and Anderson, in preparation). (B) *Cer1* was expressed at the normal position in *Rac1 epiblast-deleted* embryos. (C) In embryos where *Rac1* was deleted from the VE using *Ttr-Cre*, there was a strong constriction at the embryonic/extra-embryonic boundary (arrow) and abnormal headfolds. Axis specification, as marked by *T* expression, appeared normal at e7.5. (D) At e6.5, *Cer1* was expressed close to the distal tip of the embryo in the majority of *Rac1 VE-deleted* embryos (4/7) and had reached the extra-embryonic boundary in 3/7. (E) *T* was expressed in a ring in 3/10 *Rac1 VE-deleted* embryos at e6.5 and in a spot on the posterior side of the embryo in 7/10 embryos. (F) *Wnt3* was expressed in a ring in 2/2 *Rac1 VE-deleted* embryos at e6.5. Scale bars = 100 µm.

A number of genes, including *Cripto* and *Nodal*, are required in the epiblast for AVE migration [34]–[36]. To test whether Rac1 acts in the epiblast to promote AVE migration, we crossed animals carrying the *Rac1* conditional allele (*Rac1^cond^*) with mice that carry the *Sox2-Cre* [37] to remove *Rac1* from the epiblast but not the VE (we refer to these embryos as *Rac1 epiblast-deleted*). In contrast to the early lethality of the null embryos, *Rac1 epiblast-deleted* embryos survived until e8.5. The *Rac1 epiblast-deleted* embryos had a single AP axis, as marked by expression of *Brachyury* (Figure 3A), although they subsequently showed a variety of later morphogenetic defects (Migeotte and Anderson, in preparation). The AVE migrated normally in the *Rac1 epiblast*-*deleted* embryos, as shown by location of the *Cer1*-expressing cells in the proximal embryonic region at e6.5 (Figure 3B). Thus Rac1 is not required in the epiblast for axis specification or AVE migration.

To test whether Rac1 is required within the cells of the VE for their migration, we carried out the reciprocal experiment in which we deleted *Rac1* in the VE using a Cre driven by the transthyretin promoter (*Ttr*-*Cre*) [38]. *Ttr-Cre* is expressed in all VE cells by e5.5 [38], although a Cre reporter line showed that the expression was not uniform among cells at e5.5 and became stronger by e6.0. *Rac1^cond^*; *Ttr-Cre* embryos (*Rac1 VE-deleted*) survived longer than *Rac1* null embryos, to as late as e8.25. At e7.5, they displayed a constriction between embryonic and extra-embryonic regions and the headfolds had abnormal morphology (Figure 3C). Marker analysis showed that the initial specification of the AP body axis was defective in the majority of *Rac1 VE-deleted* embryos: *T* (Figure 3E) and *Wnt3* (Figure 3F) were expressed at e6.5 in a ring at the embryonic/extra-embryonic border, as in the null embryos.

**Figure 4:**
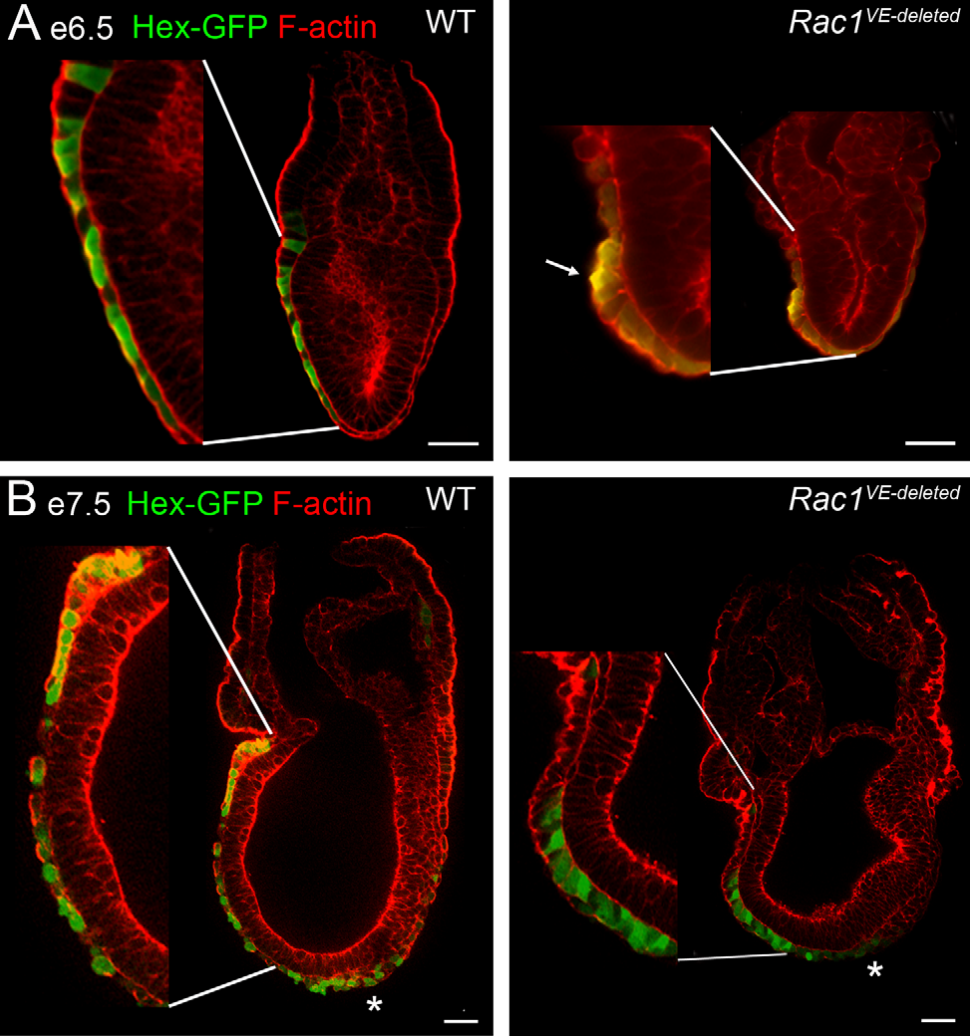
Epithelial organization of *Rac1 VE-deleted* embryos. Individual confocal sections of whole-mount embryos stained for F-actin (red). AVE cells were marked with the Hex-GFP transgene (green, staining with anti-GFP antibody in A and native GFP in B). After AVE migration is complete, at e6.5 (A) and e7.5 (B), AVE cells formed a single-layer epithelium and were squamous in wild-type embryos, with the exception of the most proximal cells, which were cuboidal. At e7.5, definitive endoderm cells expressing Hex-GFP (marked by an *) were present at the distal end of the primitive streak. In VE-deleted embryos, AVE cells had failed to reach the embryonic/extra-embryonic boundary. The Hex-GFP-expressing cells retained the columnar shape characteristic of migrating AVE cells and displayed a strong apical actin at e6.5 (arrow in A). Scale bars = 50 µm. Insets are 2×.

AVE migration was disrupted in the *Rac1 VE-deleted* embryos. At e6.5, *Cer1* was expressed close to the distal tip of the embryo in ∼50% of *Rac1 VE-deleted* embryos (Figure 3D), indicating defects in AVE migration. At cellular resolution, the distribution of Hex-GFP expressing cells showed that AVE migration was abnormal in most *Rac1 VE-deleted* embryos, when assayed at e6.5 or e7.5 (Figure 4A,B). However, most AVE cells had moved away from the distal tip of the embryo in the majority of e7.5 *Rac1 VE-deleted* embryos (Figure 4). The milder defects in AVE migration and axis specification seen in the *Rac1 VE-deleted* embryos compared to the nulls were probably due to the perdurance of Rac1 protein after the relatively late expression of the *Ttr-Cre* transgene. In contrast to the abnormal expression of streak markers at e6.5, the partial migration of the AVE in *VE-deleted* embryos correlated with the specification of an AP axis at e7.5, as shown by staining for *Brachyury* (Figure 3C); a similar correction of earlier defects has been seen in other mutants that disrupt AVE migration (*Otx2* [30]; *Nap1* [10]). Nevertheless, the dependence of AVE migration on the presence of *Rac1* in the VE, but not in the epiblast, demonstrates that Rac1 acts autonomously within the VE to promote migration of the AVE.

### Global Epithelial Organization of the VE Is Normal in Rac1 Mutants, but Cell Shape Is Affected by the Loss of Rac1

**Figure 5:**
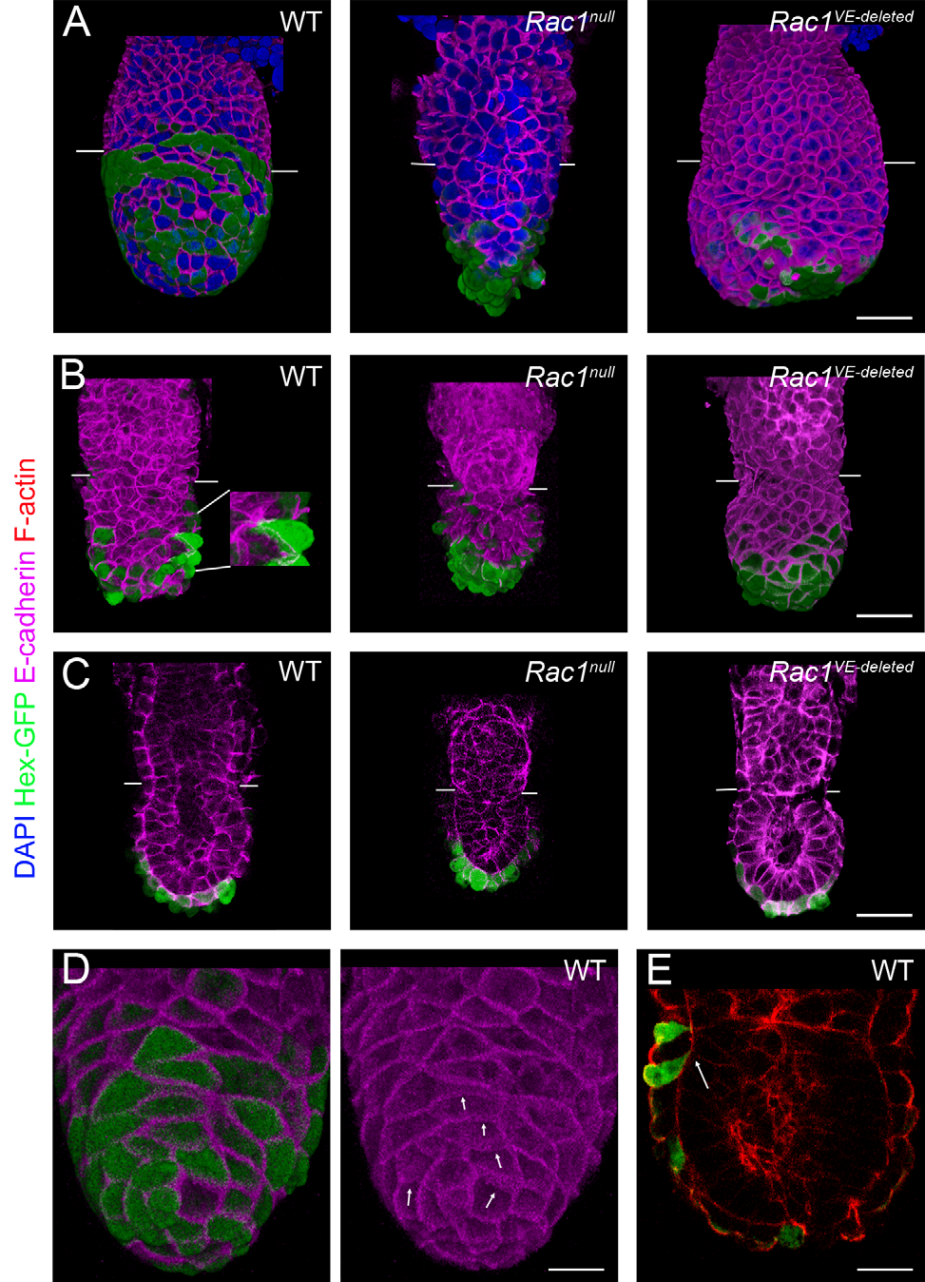
E-cadherin during migration. (A, B) 3D reconstructions showing expression of Hex-GFP (green, staining with anti-GFP antibody) and E-cadherin (magenta). See Figure S2 for the separated E-cadherin channel. (A) Embryos at the time when migration should be nearly completed. Some wild-type AVE cells have reached the extra-embryonic boundary (marked by white lines in all embryos), while they remain distal in stage-matched *Rac1 null* and *Rac1 VE-deleted* embryos (dissected at e6.25). (B) e5.5–e5.75 embryos during the time of AVE migration. Wild-type and mutant Hex-GFP cells had comparable levels of E-cadherin. Migrating wild-type embryonic VE cells had diverse shapes, and some cells with long projections could be observed (inset, 2×). In *Rac1* null embryos, cells were more regular and rounder. *Rac1 VE-deleted* embryos had a variable phenotype at e5.75. Some mutants were indistinguishable from wild-type (5/14), some had AVE cells that migrated from distal to proximal but failed to spread laterally (5/14), and some (shown) displayed a partial distal to proximal migration (4/14). (C) Individual sections from Z-stacks of e5.5–e5.75 embryos, stained for E-cadherin. Wild-type AVE cells retained adherens junctions during migration. E5.5 *Rac1 null* and *Rac1 VE-deleted* embryos displayed a single-layered epithelium with normal-appearing adherens junctions. (D) 3D reconstructions of Z-stack of a wild-type e5.5 embryo. The lateral membrane of some cells was at an oblique angle to the basement membrane, causing the E-cadherin staining to appear fuzzy (arrows). (E) Individual section from a Z-stack of a wild-type e5.75 embryo stained with phalloidin to visualize F-actin (red). AVE cells on the proximal anterior surface of the embryo are tilted in the apical/basal plane, so that their basal surfaces (arrow) are more anterior than their apical surface. Scale bars = 50 µm in A to C, and 25 µm in D and E.

We confirmed that wild-type AVE cells retain E-cadherin-positive adherens junctions during the time of migration (Figure 5 and Figure S3) [39]. Tight junctions, visualized by ZO1 expression, were present between wild-type AVE cells and between AVE cells and other cells in the VE during (Figure S4A) and after migration (Figure S4B and C). Apical-basal polarity was also retained during migration, as wild-type migrating AVE cells contacted the basement membrane at all times and expressed ZO1 exclusively at their apical surfaces (Figure S4A).

Because Rac-dependent modulation of adherens junctions is thought to play a role in cell migration and rearrangements [40], we examined the organization of the VE epithelium in *Rac1* mutants. Adherens junctions (marked by E-cadherin, Figure 5) and tight junctions (marked by ZO1, Figure S4) were present in the mutant embryos at e5.5, and expression of these junctional markers was indistinguishable from wild-type. Apical-basal polarity was also normal in the mutant VE at e5.5 (not shown). Between e5.5 and e5.75, some mutant AVE cells lost contact with the basement membrane (Figure S4A), prefiguring the distal AVE cell clustering we observed in most e6.5 *Rac1* null embryos (Figure S2).

Other aspects of VE organization appeared normal in *Rac1* mutant embryos: phospho-histone H3-positive mitotic cells were present in the *Rac1* mutant VE, and little apoptosis was observed in the VE layer, although there was large-scale apoptosis in the epiblast of *Rac1* null embryos at e6.5 (not shown). The VE is required to transport nutrients to and waste from the early embryo [41], but *Rac1 VE-deleted* embryos were comparable in size to wild-type littermates and showed normal epithelial organization at e6.5 and e7.5 (Figure 4). Thus global aspects of epithelial organization of the VE do not depend on Rac1 and are unlikely to be responsible for defects in AVE migration.

**Figure 6:**
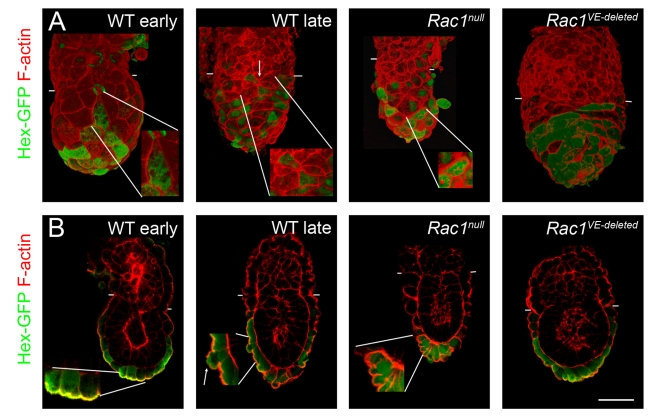
F-actin during migration. (A) Anterior views of 3D reconstructions of Z-stacks of e5.5–e5.75 embryos expressing Hex-GFP (green, staining with anti-GFP antibody), stained for F-actin (red). The embryonic/extra-embryonic boundary is marked by white lines in all embryos. The early e5.5 wild-type embryo was cultured for 1 h prior to fixation; these conditions favored the preservation of long protrusions. Wild-type embryonic VE cells had diverse shapes during migration (inset, 1.5×), while in *Rac1* null (inset, 1.5×) and *Rac1 VE-deleted* embryos, cell shapes were more regular and rounder. Wild-type VE cells formed vertices of up to 7 cells (arrow, inset, 1.5×). (B) Individual frontal sections from Z-stacks of e5.5–e5.75 embryos expressing Hex-GFP (detected with anti-GFP antibody). F-actin staining was weaker in the apical surface of migrating cells (arrow), while in wild-type pre-migration cells and in mutant cells apical F-actin staining was retained (insets, 2×). Scale bars = 50 µm.

E-cadherin staining showed that VE cells throughout the embryonic region of wild-type embryos had a range of cell shapes (Figure 5A,B; Figure S3). In the extra-embryonic portion of e5.5–e6.0 embryos, most VE cells were regularly packed in a hexagonal array; in contrast, the VE cells in the embryonic region had irregular cell shapes, frequently with only 3–4 sides (Figure S5) and some multicellular rosette-like structures with vertices of 6 or more cells were observed (Figure S3A, Figure 6A). High-resolution static images revealed that some AVE cells showed protrusions characteristic of migrating cells (Figures 5B and 6A). In contrast, *Rac1* mutant cells were more uniform in shape (Figure 2, Figure 5A and B, Figure 6A).

While there was strong E-cadherin expression between neighbors in most of the wild-type VE, we observed more diffuse E-cadherin expression on some faces of migrating Hex-positive cells (Figure 5D). Careful examination of Z-sections in embryos where the direction of migration could be predicted showed that the apparently weaker staining corresponded to cell boundaries that were tilted along the apical-basal axis, such that the proximal side of the cell (the presumptive leading edge) was basally located and partially covered by the cell in front (Figure 5D and E). In contrast, expression of E-cadherin appeared uniform on Hex-positive *Rac1* mutant cells (Figure 5B and Figure S3B), consistent with their failure to migrate. Thus *Rac1* mutant AVE cells fail to undergo the cell-shape changes that accompany migration.

### F-Actin Organization During AVE Migration Depends on Rac1

The organization of F-actin revealed by phalloidin staining, like the E-cadherin staining, showed differences in cell shapes between wild-type and mutant VE cells. 3D reconstructions of wild-type embryos stained for F-actin highlighted the variety of cell shapes among embryonic VE cells (Figure S5), as well as the presence of multicellular rosettes (Figure 6A). In contrast, *Rac1* mutant cells were uniform and rounded (Figure 6A).

Because our studies were motivated by the hypothesis that Rac1 acts upstream of the WAVE complex to reorganize F-actin during AVE cell migration, we examined the organization of F-actin in migrating VE cells. Individual sections from Z-stacks showed that most wild-type VE cells, and pre-migration AVE cells, had strong cortical actin. In contrast, some migrating AVE cells had weaker apical F-actin, probably reflecting dynamic F-actin rearrangements in those cells (Figure 6B, arrow). Both Hex-GFP-positive and -negative *Rac1* mutant VE cells showed strong cortical actin (Figure 6B, Figure 4), consistent with a requirement for Rac1 in the dynamic rearrangements of the actin cytoskeleton required for migration.

### AVE Migration Is Coordinated, Involves Neighbor Exchange, and the Extension of Long Lamellar Protrusions; All Aspects Are Rac1-Dependent

**Figure 7:**
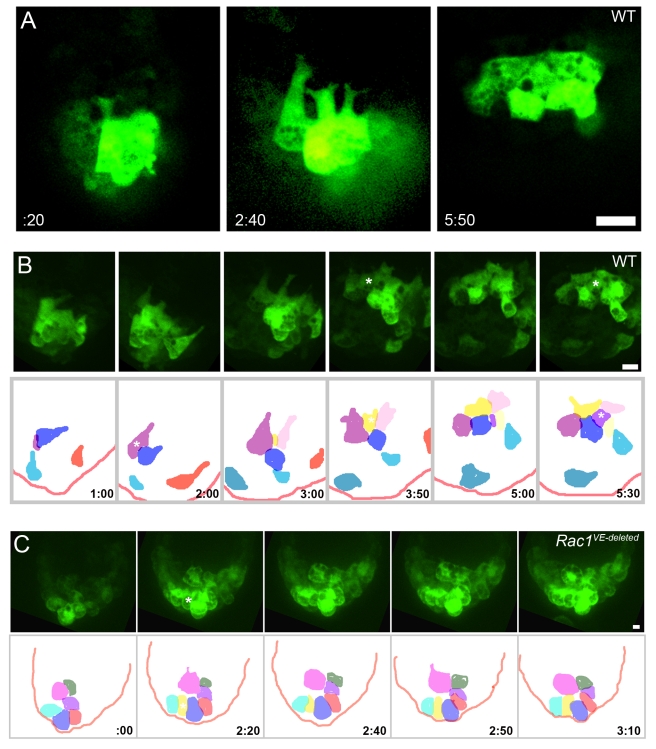
AVE cells send long lamellipodia that are spatially and temporally coordinated and depend on Rac1. Hex-GFP expressing embryos were dissected at e5.5 and cultured for up to 18 h. Proximal is up in all panels. (A) Individual confocal sections extracted from an 18 h live imaging experiment (Video S1). Four Hex-positive cells simultaneously sent long, parallel projections toward the extra-embryonic region. By 5 h 50 min, the cells reached the boundary of the extra-embryonic region, and the protrusive activity stopped. Images were aligned relative to the distal border of the embryo. (B) Hex-GFP-positive cells migrated from distal to proximal in a coordinated fashion, and then spread laterally on the anterior surface of the embryo. In the lower panel, cells from maximal projection images from stacks of confocal sections (stills from Video S1) were colored to follow individual cell movements and changes of neighbors. Asterisks show the intercalations of previously hidden cells. (C) In *Rac1 VE-deleted* embryos there was no cell migration, and no exchange of neighbors (lower panel). Asterisk shows the intercalation of a previously hidden cell. In the lower panel, cells from maximal projection images from stacks of confocal sections (stills from Video S11) were colored. Scale bars = 30 µm.

**Figure 8:**
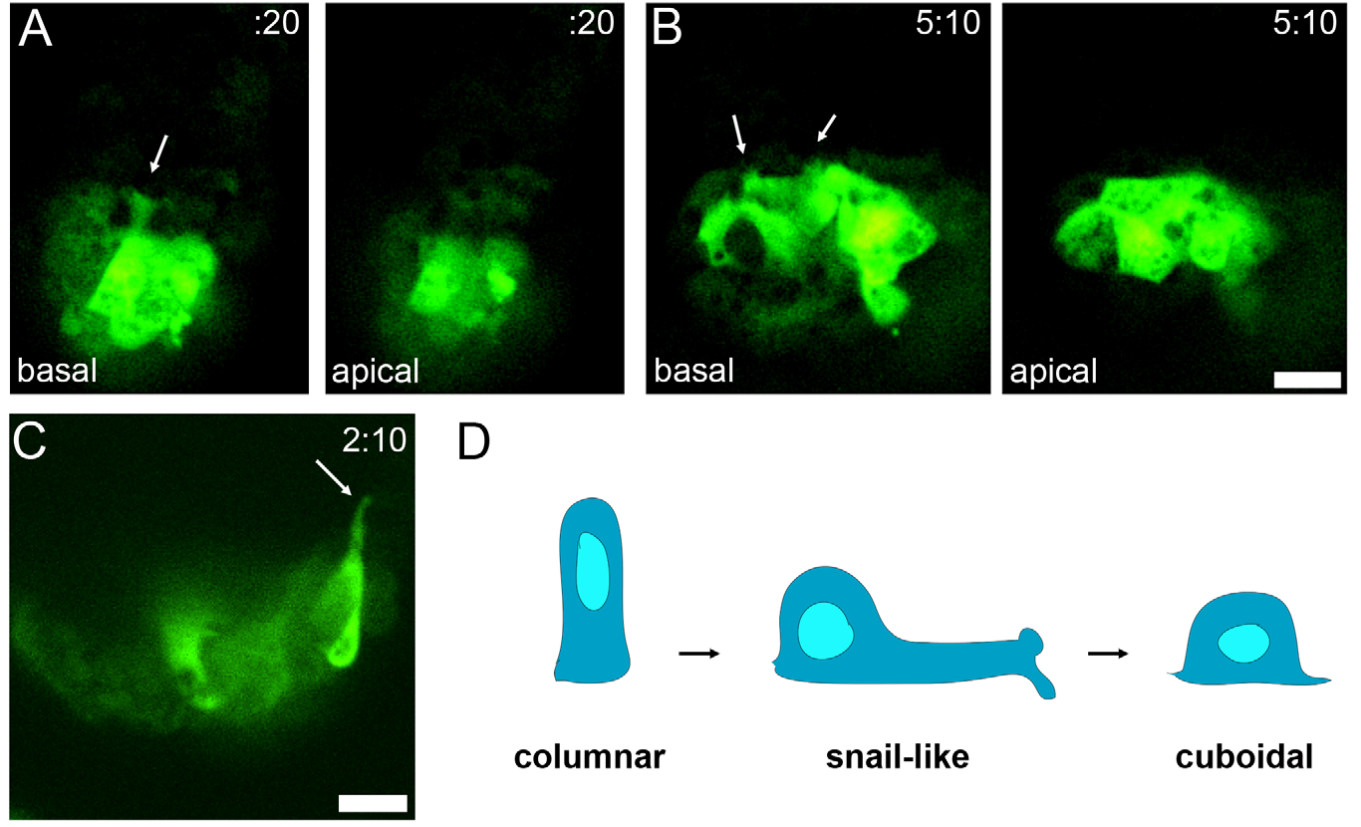
AVE cells insert basal projections between neighboring cells to move forward. (A) Individual confocal sections of a migrating snail-like AVE cell (dark blue cell from Video S1). The basal section shows a long lamella with two projections at its end (arrow); the projection is not visible apically. The distance between the apical and basal optical sections is 8 µm. (B) Individual confocal sections of an AVE cell (yellow cell from Video S1) that has arrived at the embryonic/extra-embryonic border. The cell shape became cuboidal. The basal surface was marked by retracting protrusions (arrow) that are not visible in the apical plane. The distance between the apical and basal optical sections is 14 µm. Scale bar = 30 µm. (C) Individual confocal section of an AVE cell from Video S1, oriented to show the apical-basal profile of the cell. The long projection is on the basal side. (D) Schematic of the shape changes of the cells with long lamellar projections. At e5.5, AVE cells are columnar. During migration, cells acquire a snail-like morphology with a round cell body and a highly elongated lamella ended by two lateral horn-like protrusions. At the end of migration, the cells become cuboidal.

We used time-lapse video microscopy to compare the behaviors of migrating AVE cells in cultured wild-type and mutant embryos (Figure S6). As described previously [2], wild-type Hex-GFP cells in the early phase of migration extended transient protrusions directed towards the proximal region of the embryo (Figure 7A and B, Figure 8A; Videos S1, S2, and S3).

Live imaging revealed dramatic dynamic cellular behaviors that were not captured by static images. In two time-lapse experiments in which we could follow AVE migration from beginning to end, we saw that 5–7 leading Hex-positive cells extended parallel long lamellar protrusions toward the proximal region of the embryo (Videos S1 and S2). Long projections were present for about 3 h (Figure 7B, Video S1). The vectors of movement of individual cell bodies were highly oriented towards the proximal region of the embryo (Figure S7).

In most embryos there were non-green cells between Hex-GFP+ cells. The GFP-negative cells may have corresponded to AVE cells that did not express the transgene [2] or AVE cells may have moved in smaller groups to navigate through the VE epithelium. Cell tracking showed that AVE cells exchanged neighbors during migration (Figure 7B, Video S1). Although all Hex-GFP cells belonged to the single-layered VE epithelium, some with small apical surfaces were mostly hidden by their more spread neighbors and became visible at the apical surface of the epithelium during the observation period (marked by * in Figure 7B, Video S1), highlighting the shape changes that occur during migration. We observed no more than two mitoses in AVE cells per embryo during the migration period, which makes it unlikely that cell division contributed to cell intercalation. Approximately 5 h after the appearance of long forward projections, the leading Hex-GFP cells arrived at the embryonic/extra-embryonic border. They ceased forward movement, sent shorter lateral projections, and spread laterally on the anterior side of the embryo (Figure 7A and B, Figure 8B, Videos S1 and S2). After migration, AVE cells became more dispersed on the anterior surface, probably due to the growth of the embryo.

Analysis of the Z-planes for individual cells (Figure 8A and B), 3D reconstruction (Video S4), and analysis of cells captured in a profile view (Figure 8C) showed that the long projections were located on the basal side of cells, giving cells the appearance of a snail (Figure 8D). The tight packing of trailing GFP-positive cells that did not have an edge with non-GFP-cells made it difficult to assess the cell contours with certainty. However, the trailing cells that we could observe in detail displayed shorter projections that were also on the basal side (Figure S8 and Video S5).

**Figure 9:**
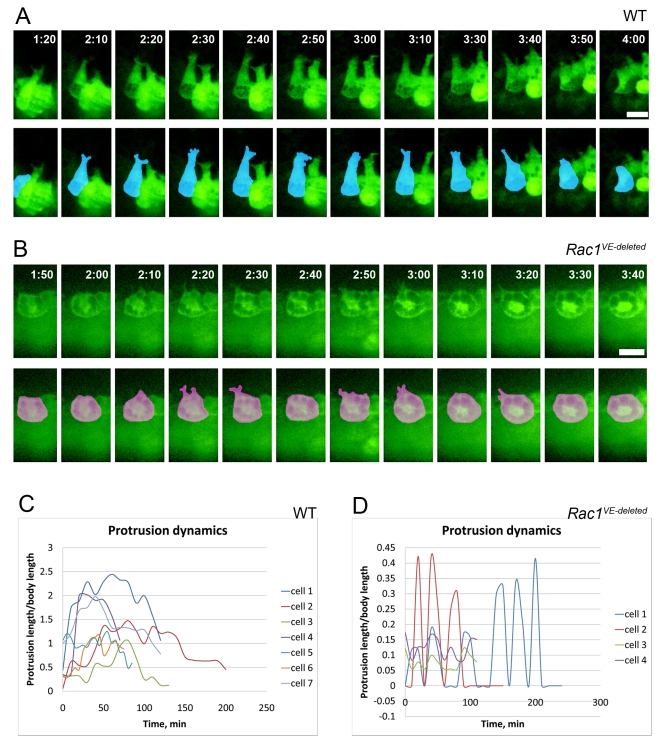
The long stable protrusions of AVE cells are Rac1-dependent. (A, B) Individual confocal sections were aligned against the distal border of the embryo (A) or against the cell body (B), and painted to highlight cell shape in the lower row. (A) The wild-type cell displayed is the purple cell in Figure 7B. The protrusive activity persisted for 2 h. The long protrusions had a stable direction for about 30 min (2 h 10 min–2 h 40 min), then retracted partially and extended at a slightly different angle (3 h 10 min–3 h 40 min) (Videos S1 and S6). (B) The *Rac1* mutant cell displayed is the pink cell in Figure 7C. The cell was non-polarized and rounded. The small protrusions of mutant cells were unstable (Videos S11 and S13). Scale bar = 30 µm. (C, D) Quantification of protrusion length and stability. (C) In wild-type embryos, the protrusions lasted for at least 1 h and their length reached up to 2.5 times the diameter of the cell body (defined as the circular region of the cell surrounding the nucleus). 7 cells from 5 experiments were analyzed. (D) In *Rac1 VE-deleted* embryos protrusions (4 cells from 2 experiments) were short (less than half the cell body diameter) and transient (∼10 min).

Morphometric analysis of protrusion dynamics suggested that the long lamellar protrusions on the leading Hex-expressing cells might probe the path that the cells would follow. Most long projections extended, then partially retracted, and extended again at a slightly different angle, keeping the same global direction (Figures 9A and S9; Videos S1, S2, S3, S6, and S7). Individual lamellar protrusions lasted for 1–2.5 h and their maximal length was 2.5 times as long as the diameter of the cell body (Figures 9C and S10, Video S8). The lamellar projections ended in two smaller lateral filopodia-like protrusions that extended and collapsed more dynamically than the lamella as a whole (Figures 9A and S9; Videos S3, S6, and S7). While the long projections were extended, the cell bodies displayed pulsing contractions with little net movement, after which the cell body moved rapidly following the direction of the projection (Video S3).

Live imaging of a *Rac1* null embryo from e5.75 to e6 made it possible to observe the formation of the grape-like cluster at the embryo tip. Some mutant AVE cells located at the distal tip acquired a pear shape, with a smaller basal surface, and lost contact with the basal membrane, but did not shed off the embryo, presumably due to the acquisition of new cell-cell contacts (Figures S2 and S11, Videos S9 and S10).

Live imaging of *Rac1 VE-deleted* embryos confirmed that Rac1 is required for the cell shape changes and the formation of the protrusions that accompany cell migration (Figure 7C, Video S11). No active movement of Hex-GFP-positive cells was observed. Although the growth of the embryo appeared normal, very little reorganization of cells within the epithelium occurred: we observed no neighbor exchange or cell shape changes. Mutant cells were round (Figure S10, Video S12), and protrusions observed in a low proportion of Hex-GFP-positive cells were short and transient. In the two *Rac1 VE-deleted* mutants we imaged for a long period, we found two AVE cells per embryo that extended short protrusions (less than half the cell body size) that lasted only 10–20 min (Figure 9B and D, Video S13). Thus Rac1 is required for both cellular events that accompany the coordinated epithelial migration of AVE cells: the extension of long processes and the reorganization of the epithelium.

## Discussion

### Rac1 Is Required for Establishment of the AP Body Axis of the Mouse Embryo

Embryological and genetic studies have shown that migration of the AVE cells is crucial for the establishment of the AP axis of the mouse embryo [4]. Although genetic studies in the mouse have defined requirements for the Wnt and Nodal pathways in establishing the competence of the AVE cells to migrate, remarkably little is known about the signals or cellular mechanisms that direct migration of these cells. Here we have demonstrated that Rac1, a key regulator of cell movement, is absolutely essential for the establishment of the AP axis of the mouse embryo.

In the absence of Rac1, all cells in the proximal epiblast acquire a posterior identity and no AP axis is defined. This profound disruption of AP patterning suggested that there might be an earlier defect in AVE migration. Although Rac1 is required for survival and adhesion of the mesoderm ([25]; Migeotte and Anderson in preparation), we find that Rac1 is not required for epithelial organization, cell survival, or proliferation in the VE. Wnt signaling is required for specification of the AVE [42]. It has been argued that Rac1 is required for canonical Wnt signaling [19]; in contrast, the AVE was specified normally in *Rac1* null embryos, as marked by the expression of *Cer1* and *Hex*. We find, however, that Rac1 is specifically and absolutely required for migration of AVE cells.

### Rac1 Acts through the WAVE Complex to Regulate Collective Migration of the AVE

We showed previously that mutations that disrupt Nap1, a component of the WAVE regulatory complex, cause duplications of the AP axis with ∼25% penetrance, associated with the failure of normal migration of AVE cells [10]. Our findings support our prediction that Rac1 acts upstream of Nap1 to control directed cell migration of the AVE. Our previous studies did not test whether Nap1 was required in the migrating cells themselves. Here we were able to use a conditional allele to show that Rac1 is required in the VE, and not in the epiblast, for migration of AVE cells.

Recently we have characterized a stronger allele of *Nap1* caused by a gene trap insertion, *Nap1^RRQ^*. The *Nap1^RRQ^* phenotype is intermediate between the *Nap1^khlo^* and *Rac1* null phenotypes. AVE migration is disrupted in half of *Nap1^khlo^* embryos [10] but is disrupted in all *Nap1^RRQ^*, which leads to defects in AP axis specification in all mutants (Figure S12). The *Nap1^RRQ^* allele is not a null allele, as some wild-type transcript is present in mutant embryos due to splicing around the gene trap insertion (not shown). Nevertheless, the strong phenotype of the *Nap1^RRQ^* allele highlights the importance of the WAVE complex in AVE migration and argues strongly that one of the principle activities of Rac1 in AVE migration is to control activation of WAVE.

Our analysis of cellular behavior during AVE migration is also consistent with the central role of the WAVE complex. During wild-type AVE migration, the leading cells extend very long, lamellipodia-like projections (the type of structures that depend on WAVE) that are completely dependent on Rac activity. *Rac1 VE-deleted* embryos, which have decreased Rac1 activity in the VE, show a preferential loss of lamellipodia, although they can extend a few short projections. Our findings suggest that AVE migration is driven by an extracellular signal that activates Rac1 in the AVE cells to direct them to extend proximal, WAVE-dependent lamellar projections.

In contrast to the variable AVE migration defects seen in *Nap1* mutants, 100% of *Rac1* null mutants display no migration at all. Because the characterized alleles of *Nap1* are not null, it is not known whether the complete loss of Nap1 would cause a stronger defect in AVE migration. However, the stronger phenotype caused by loss of *Rac1* raises the possibility that Rac1 has roles in the AVE in addition to the regulation of the WAVE complex.

Rac1 could for example influence movement of AVE cells through regulation of the dynamics of adherens junctions within the VE [43]–[45]. Although wild-type migrating AVE cells retain E-cadherin between all neighbors during migration, the neighbor exchanges we observe indicate that junctions must be dynamically remodeled during migration. There is no global loss of junctions in the mutants, as *Rac1* VE cells express adherens and tight junction proteins at comparable levels to wild-type cells. Nevertheless, *Rac1* mutant VE cells at the onset of migration appear to be rounder than wild-type cells: they lack the tight packing seen in wild-type and their apical surfaces are dome-shaped. During migration, the shapes of both Hex-GFP positive and Hex-GFP negative cells within the embryonic VE become irregular. In contrast, cell shapes in the VE of *Rac1* null mutants remain regular. These findings suggest that Rac1 could contribute to the mechanical forces within the VE epithelium that favor migration in the wild-type embryo.

### Residual AP Asymmetry in Some Rac1 Mutants

There has been controversy in the field concerning a possible role for asymmetries in the blastocyst in the establishment of the AP body axis [42],[46]–[48]. We found that although most *Rac1* mutants expressed markers of the primitive streak radially around the embryonic circumference, 30% of null embryos examined showed some asymmetry in the expression of streak markers. In these embryos, the cluster of distally located DVE/AVE cells was slightly skewed away from the side of streak marker expression, suggesting that some aspect of AP asymmetry might be independent of AVE migration. If these skewed DVE cells secrete Wnt and Nodal inhibitors, that might account for the residual asymmetry in *Rac1* mutants. However, in the majority of *Rac1* null embryos, this shift is either too small to be detected or nonexistent and does not lead to posterior restriction of streak markers. We therefore conclude that there may be a small AP bias derived from the blastocyst embryo, but that it is not detectable in most embryos and that any early bias must be reinforced through cell migration.

### Rac1 Is Required for the Collective, Epithelial Migration of the AVE

Collective cell migration, in which cells migrate while retaining epithelial organization, is common in both invertebrates and vertebrates [49]–[51]. One common feature of collective migration is that cells at the leading edge display some characteristics typical of mesenchymal cells, such as the presence of highly dynamic actin-rich cellular protrusions, while retaining junctions with their neighbors. This cohesive mode of migration allows cell guidance as well as mechanical coupling and shaping of tissues.

The live imaging experiments made it possible to define the cellular behaviors of the wild-type AVE during migration, which, together with our static analysis, suggested that AVE movement has the properties of collective epithelial migration. Although the Hex-expressing cells do not form a single coherent group, clusters of AVE cells insert basal projections between neighboring cells and pull themselves forward through the epithelium, while retaining junctions amongst themselves and with adjacent cells, as well as with the basal membrane. The leading AVE cells change shape from columnar to cuboidal and send long extensions that can extend several cell diameters ahead of the cell body. The long protrusions on the leading cells are unusual in structure: they have two smaller protrusions at their tips. The long protrusions are stable, lasting 1–2 h, similar to the long cellular extensions found in many cells that move in a directional manner during development [52], such as *Drosophila* border cells [53] and tracheal cells [54]. These stable projections are very different from short-lived lamellipodia found in cells that follow rapidly changing gradients such as neutrophils. We suggest that these unusual properties may facilitate the reception of signals that guide the direction of AVE migration.

All the features of AVE migration were lost in *Rac1* mutant embryos: null mutant cells failed to extend any projections, and no modulation of junctions or no neighbor exchanges were observed. The behavior of *Rac1* mutant AVE cells is very different from what has been observed in *Rac1* null fibroblasts, which migrate as rapidly as wild-type cells in response to PDGF by extending pseudopodia-like extensions and do not depend on lamellipodia or membrane localization of Arp2/3 for movement [55]. The difference between the two cell types suggests that migration within an epithelium may be more dependent on Rac1 than is the migration of mesenchymal cells. Rac has previously been shown to be important for collective migration in *Drosophila* in the border cells of the ovary [56],[57] and during tracheal branching [40]. Collective cell migration is likely to be a primary mode of tissue movement in mammalian branching morphogenesis [58] and tumor invasion [51]; our findings on the AVE raise the possibility that Rac1 is also a crucial regulator of these types of mammalian collective migration.

## Methods

### Mouse Strains and Genotyping

A *Rac1* conditional allele [26] was crossed to mice expressing *CAG-Cre* [59] to generate a null allele. The *Rac1* conditional allele was analyzed in a C3H background and the null allele was analyzed on a mixed C3H/CD1 background. *Sox2-Cre* [37] and *Ttr-Cre* [38] were used to delete the gene in the epiblast and VE, respectively. Epiblast-deleted embryos were of the genotype *Rac1 floxed/null; Sox2-Cre/+*, and VE-deleted embryos were of the genotype *Rac1 floxed/null; Ttr-Cre/+*. An ES cell line (RRQ139) carrying a gene trap insertion downstream of exon 6 of the *Nap1* gene was made by BayGenomics (http://www.mmrrc.org/). This gene trap should produce a fusion of the first 297 amino acids of Nap1 and β-geo. Mice derived from the ES cell clone were kept on a C3H background and are referred to as *Nap1^RRQ^*. The *Foxa2* allele [60] was analyzed on a mixed CD1/C3H background. The *Hex-GFP* line was previously described [2]. The BAT-gal line was characterized in [61].

### Analysis of Mutant Embryos

Embryos at e6.5 and older were dissected in PBS 0.4% BSA; e5.5 embryos were dissected in PB1 [62]. In situ hybridization and X-gal staining were carried out as described [63]. Whole-mount embryos were imaged using a Zeiss Axiocam HRC digital camera on a Leica MZFLIII microscope. For immunofluorescence, embryos were fixed in 4% paraformaldehyde (PFA) in PBS for 1 to 3 h on ice and washed in PBS. Fixed embryos were embedded in OCT and cryosectioned at 8 µm thickness. Staining was performed in PBS, 0.1% Triton, 1% heat-inactivated goat serum for e6.5 and e7.5 embryos, and as described in [39] for e5.5 embryos. Whole-mount embryos were imaged on a Leica DM1RE2 inverted confocal. Confocal datasets were analyzed using the Volocity software package (Improvision Inc.).

### Antibodies

Antibodies were: rat anti-E-cadherin, 1∶500 (Sigma); rabbit anti-GFP, 1∶500 (Invitrogen); chick anti-GFP, 1∶500 (Abcam); rabbit anti-ZO1, 1∶200 (Zymed). Rac1 antibodies showed background staining, which made it impossible to directly measure the level of residual Rac1 protein in the VE of e5.5 embryos. F-actin was visualized using 10 U/ml TRITC-Phalloidin (Molecular Probes), and nuclei using DAPI (Sigma). Secondary antibodies were from Invitrogen.

### Embryo Culture

Embryos were cultured in 50% DMEM-F12 with HEPES and L-glutamine without phenol red (Gibco), 50% rat serum (Taconic or Harlan) in a dome covered with mineral oil. Imaging was performed using a spinning disk Perkin Elmer UltraVIEW VoX Confocal Imaging System. Nikon Eclipse Ti microscope was equipped with Nikon Plan Fluor 20×, 0.5NA or Plan-NEOFLUAR 40×/1.3 NA, with Hamamatsu EM-CCD C91100-13 (high frame rate format of 512×512 pixels), Hamamatsu C9100-50 (Japan), or Andor iXonEM+EMCCD Camera (Belfast, Northern Ireland) and LiveCell TM temperature-controlled stage (Live Cell Pathology Devices Inc.). Acquisition was controlled by Volocity 5.0.3 Build 4 software or MetaMorph (Molecular Devices). 488 nm laser power was set to 2%. Exposure time was in a range of 400–800 ms. Acquisition frequency was set for 6 time points per hour.

Epifluorescence time-lapse video microscopy was performed on Axiovert 200 M equipped with LD Plan-NEOFLUAR 0.4NA 20× lens, Hamamatsu C4742-80 Orca-ER, and temperature-controlled stage. Acquisition was controlled by Axiovision software.

### Image Analysis

Images from the live imaging experiments were calibrated, aligned, and adjusted for digital contrast using MetaMorph (Molecular Devices). All measurements were performed using MetaMorph and all data were transferred to Excel (Microsoft) for analysis and representation. Manual alignment of images was performed using Align Stack function (MetaMorph). To analyze migration of AVE cells, we used the distal border of the embryo as a reference point to align images. To monitor protrusive activity of individual cells, alignment was performed against the distal border of the embryo or the center of the cell body. To monitor behavior of individual AVE cells, we manually highlighted cells and the embryo distal border in shades of different colors (50% opacity) using Adobe Photoshop. Maximal projections or images of individual optical sections were used to determine the outline of cells. Towards the end of experiments, when the signal became saturated because the expression of the transgene increased, we used the cell shape of previous time frame assuming that the cell shape did not change significantly. Relative protrusion length was determined by ratio of the protrusion length over the diameter of the cell body. The diameter of cell body was defined as double the distance from the center of the nucleus to the posterior cell border. For the determination of the cell shape factor, we used high resolution Z-stacks of live embryos (step size 0.2–0.5 µm) and chose candidate cells with obvious protrusions. We used multiple optical sections to outline the cell perimeter (Region tool, Metamorph). Thresholded images were used to calculate the shape factor (shape factor = 4πA/P^2^), which is calculated from the perimeter (P) and the area (A) of the object (the cell) using the Integrated Morphometry tool (Metamorph). 1 corresponds to a perfect circle and values <1 represent progressively more elongated or irregular shapes.

## Supporting Information

Figure S1 **Gastrulation initiates in** ***Rac1^null^*** **embryos.** Single confocal sections of e7.5 embryos stained with phalloidin to visualize F-actin (red). Mutant embryos generate mesoderm, which is present around the embryo between the epiblast and endoderm layers. There are numerous pyknotic nuclei in the mesoderm layer, as previously described [25]. Scale bars = 50 µm.
(4.71 MB TIF)

Figure S2 **AVE cells that fail to migrate form a multilayered epithelium.** Single confocal sections of e6.5 embryos stained for E-cadherin (magenta). At e6.5, AVE cells remained at the distal tip of the embryo in *Rac1*, *FoxA2*, and *Nap1^RRQ^* mutants expressing Hex-GFP (staining with anti-GFP antibody for wild-type, *FoxA2*, and *RRQ* embryos and native GFP for *Rac1 null* embryos). AVE cells formed several rows (arrows) and were linked by adherens junctions. In *FoxA2* mutants (C), cells failed to express Hex-GFP but could be recognized through their columnar morphology (*). *Nap1^RRQ^* embryos (D) displayed a more severe phenotype than the *Nap1^khlo^* allele described in [10]. Scale bars = 50 µm.
(7.97 MB TIF)

Figure S3 **E-cadherin during migration.** E-cadherin staining from the panels A and B of Figure 5. (A) Rosette-like structures can be detected in the wild-type VE (pseudo-colored). (B) The arrow points to the extremity of the long projection seen in Figure 5B.
(6.73 MB TIF)

Figure S4 **ZO1 during migration.** (A) Individual confocal sections of e5.5–e5.75 embryos expressing Hex-GFP (green, detected with anti-GFP antibody) stained for ZO1 (magenta). In wild-type embryos, AVE cells retained apically localized tight junctions as they migrated. The VE of *Rac1* null embryos showed a normal apical restriction of tight junctions at e5.5. However, between e5.5 and e5.75, some AVE cells lost contact with the basement membrane and expressed ZO1 on their lateral surfaces, prefiguring the cluster seen in *Rac1* null embryos at e6.5 (see Figures 2 and S2). 3D reconstructions (B) and individual confocal sections (C) showing expression of Hex-GFP (green, native GFP) and ZO1 in stacks of e5.75 wild-type embryos and stage-matched *Rac1* null embryos (dissected at e6.25). Tight junctions were normal in the VE of *Rac1* null embryos at e6.25. Scale bars = 50 µm. Insets are 1.5×.
(8.24 MB TIF)

Figure S5 **Cell shape is irregular in the embryonic VE during AVE migration.** 3D reconstructions of Z-stacks of early (lateral view) and late (anterior view) embryos expressing Hex-GFP (green, native GFP), stained for F-actin (red). The embryos presented as examples were cultured for 1 h prior to fixation. A stable epithelium has a majority of pentagonal or hexagonal cells. The number of sides per cell in the extra-embryonic and embryonic regions of early and late e5.5 embryos was quantified on 3D reconstructions of Z-stacks, considering all faces of the embryos. All cells from the embryonic portion were considered, regardless of Hex-GFP expression, as all cells are likely to change shape either actively or passively. In both groups, most cells had 3 or 4 sides in the embryonic region, and 5 or 6 sides in the extra-embryonic region, and this trend was stronger in younger embryos in which the epithelium is expected to be less stable.
(6.10 MB TIF)

Figure S6 **Embryo survival and growth in culture.** Embryos dissected at e5.5 and cultured in a chamber at 37°C and 5% CO2 grow at close to normal rates under the culture conditions. Epifluorescence and phase contrast images of embryos expressing Hex-GFP at dissection and after 18 h of culture (used in Video S3). Scale bar = 100 µm.
(2.39 MB TIF)

Figure S7 **Vectors of migration of AVE cells.** Epifluorescent time-lapse images (stills from Video S3) were aligned relative to the distal border of the embryo (at the bottom in Figure). In the left panel, the lines represent the initial (blue line, middle panel) and final (green line, right panel) outlines of Hex-GFP-expressing AVE cells population. The positions of individual cells were tracked and the cumulative vectors of migration were built using the “Track Points” function of Metamorph (numbered red lines). The average migration rate was 0.12±0.01 µm/min. The vector angle (assessing the directionality of migration) was 164±4.5 degrees relative to the bottom of images. Scale bar = 30 µm.
(1.62 MB TIF)

Figure S8 **Basal projection of migrating AVE cell.** Individual confocal images (stills from Video S5) of a trailing wild-type Hex-GFP cell. The cell showed basal protrusive activity while maintaining its columnar structure. The protrusion was directed towards the embryonic/extra-embryonic border (top right) and the cell translocated over time. Scale bar = 30 µm.
(6.88 MB TIF)

Figure S9 **Front cell projection dynamics.** Individual confocal sections (stills from Video S7) were aligned relative to center of the cell body and painted to highlight cell shape. The wild-type cell displayed is the blue cell in Figure 7B. The protrusive activity persisted for about 1.5 h. Protrusions had a stable direction for about 20 min (20–40 min), then retracted partially and extended at a slightly different angle (1 h 10 min–1 h 30 min). Scale bar = 30 µm.
(6.16 MB TIF)

Figure S10 **Rac1 mutant cells fail to elongate.** The shape factor (see Methods) was calculated for cells from wild-type (12 cells from 8 embryos), *Rac1* null (4 cells from 2 embryos), and *Rac1 VE-deleted* (9 cells from 5 embryos) from high resolution Z-stacks of live embryos. A value of 1 denotes a perfect circle, and lower values represent progressively more elongated or irregular shapes. *Rac1* mutants are significantly rounder than wild type. Data are presented as mean ± SEM.
(1.19 MB TIF)

Figure S11 **Formation of the distal cluster of DVE cells in a** ***Rac1*** **null embryo.** E6.0 *Rac1* null mutant AVE cells were teardrop shaped and failed to make protrusions (stills from Video S10), and some cells appear to have lost contact with the basement membrane, which prefigures the formation of a grape-like cluster of AVE cells at the tip of the embryo. Scale bar = 30 µm.
(5.28 MB TIF)

Figure S12 **Defects in AVE migration and axis specification in** ***Nap1^RRQ^*** **embryos.** (A) 3D reconstructions of Z-stacks of e6.5 embryos expressing Hex-GFP (green, staining with anti-GFP antibody), stained for F-actin (red) or E-cadherin (magenta). In *Nap1^RRQ^* embryos, the distribution of Hex-GFP is abnormal, and many cells fail to initiate migration. Cells are round in the VE of mutant embryos, reminiscent of the *Rac1* mutant embryos. (B) Expression of *Brachyury* (*T*) at e7.5. In *Nap1^RRQ^* mutants, there appear to be multiple sites of streak initiation or, in the most severe cases, a ring of T-expressing cells in the proximal epiblast.
(10.18 MB TIF)

Video S1 **AVE cells send long lamellipodia that are spatially and temporally coordinated.** A wild-type embryo was dissected on e5.6 at mid-afternoon, and imaged for 6 h. Images were taken every 10 min. At each time point, a stack of 39 slices was taken, at 2 µm per slice. Extended focus images have been aligned to correct for the shift of the embryo, using the distal border as a reference point. Individual cells were pseudocolored to visualize their trajectories. Cells were tracked using individual confocal images of each stack to increase the accuracy of the cell contours.
(9.84 MB AVI)

Video S2 **AVE cells migrate as a group.** An e5.6 wild-type embryo was imaged for 5 h, as in Video S1. At each time point, a stack of 8 slices was taken, at 5 µm per slice. Extended focus images have been aligned to correct for movements of the embryo, using the distal border as a reference point.
(5.97 MB AVI)

Video S3 **Long projections of migrating AVE cells, visualized by epifluorescence.** A wild-type embryo was dissected on e5.6 and imaged using an epifluorescent microscope at one frame per minute for 3 h. Individual images were aligned using the distal end of the embryo as a reference point. The same type of projections seen by confocal were detected with this type of imaging. The shorter interval between images makes it possible to visualize contractions of cell bodies.
(10.09 MB AVI)

Video S4 **The projections on the leading wild-type AVE cells are located basally.** 3D reconstruction (Hex-GFP, green) from stacks of 150–200 optical sections (step size: 0.5 µm) was performed using Amira ResolveRT 4.1. The embryo outline (dotted line) was determined in Metamorph (Region outline tool) using bright field images. A single orthogonal plane of the embryo is shown in gray scale. The bounding box (orange lines) represents the volume analyzed in all the sections. The movie (from 0 to 130 degrees) includes 60 individual planes, rotated at 6 frames per second. The leading cells display a snail-like shape, with columnar cell bodies and basal projections.
(8.69 MB AVI)

Video S5 **Projections on trailing cells are located basally.** Video S5 shows a detail from Video S1. A cell initially located at the distal end of the embryo was tracked for 3 h 50 min in individual confocal sections from each time point. Frames were aligned using the center of the cell body as a reference point. The cell was pseudocolored to highlight the dynamics of projections.
(1.00 MB AVI)

Video S6 **Leading cell projection dynamics.** Video S6 shows the cell pseudocolored in pink in Video S1, which was located on the anterior surface of the embryo. It was tracked for 3.5 h in individual confocal sections from each time point. Frames were aligned using the center of the cell body as a reference point. The cell details were pseudocolored to highlight the dynamics of projections.
(2.25 MB AVI)

Video S7 **Leading cell projection dynamics.** Video S7 shows the cell pseudocolored in blue in Video S1, which was located on the anterior surface of the embryo. It was tracked for 2 h in individual confocal sections from each time point. Frames were aligned using the center of the cell body as a reference point. The cell details were pseudocolored to highlight the dynamics of projections.
(1.07 MB AVI)

Video S8 **Wild-type AVE cells are elongated.** 3D reconstruction (Hex-GFP, green) from stacks of 150–200 optical sections (step size: 0.5 µm) was performed using Amira ResolveRT 4.1. The embryo outline (dotted line) was determined in Metamorph (Region outline tool) using bright field images. A single orthogonal plane of the embryo is shown in gray scale. The bounding box (orange lines) represents the volume analyzed in all the sections. The movie (from 0 to 130 degrees) includes 60 individual planes, rotated at 6 frames per second. The movie illustrates the elongated shape and the anterior protrusion of leading AVE cells.
(8.92 MB AVI)

Video S9 **Lack of projections in a** ***Rac1*** **null embryo.** The embryo depicted in Video S9 is from a litter dissected on e5.5 at mid-afternoon and was selected because the Hex-GFP cells appeared to be round and to not have initiated migration. The embryo was smaller than its wild-type littermates at dissection but developed overnight to a degree similar to what we have observed in vivo. It was imaged from 6 pm to 10:30 am. Frames are 10 min apart. A stack of 23 slices was taken, at 2 µm per slice. The movie represents the first 5 h of imaging. It was genotyped the following day and confirmed to be *Rac1 null*.
(5.95 MB AVI)

Video S10 **Formation of a distal cluster of AVE cells in a** ***Rac1*** **null embryo.** Video S10 figures a cell from Video S9 (*Rac1* null embryo). It was tracked for 2.5 h in individual confocal sections from each time point. Frames were aligned using the center of the cell body as a reference point. The cell contour was pseudocolored to highlight cell shape changes. Some cells seem to have lost contact with the basement membrane.
(0.86 MB AVI)

Video S11 **Absence of cell movement and long cellular projections in** ***Rac1 VE-deleted*** **embryos.** The *Rac1 VE-deleted* embryo was dissected on e5.6 and imaged for 4 h 20 min. Frames are 10 min apart. A stack of 22 slices was taken, at 2 µm per slice. The embryo had developed overnight to a degree similar to wild-type embryos. Individual cells were pseudocolored in order to visualize their trajectories. The cell tracking was made using individual confocal images of each stack in order to increase the accuracy of the cell contours.
(9.57 MB AVI)

Video S12 **Cells are round in Rac1 VE-deleted embryos.** 3D reconstruction (Hex-GFP, green) from stacks of 150–200 optical sections (step size: 0.5 µm) was performed using Amira ResolveRT 4.1. The embryo outline (dotted line) was determined in Metamorph (Region outline tool) using bright field images. A single orthogonal plane of the embryo is shown in gray scale. The bounding box (orange lines) represents the volume analyzed in all the sections. The movie (from 0 to 130 degrees) includes 60 individual planes, rotated at 6 frames per second. Even the cells displaying short projections have a circular shape.
(9.72 MB AVI)

Video S13 **Projection dynamics in a** ***Rac1 VE-deleted*** **embryo.** Video S13 shows the cell pseudocolored in pink in Video S11, which is located at the distal end of the *Rac1 VE-deleted* embryo. It was tracked for 1 h 50 min in individual confocal sections from each time point. Frames were aligned using the center of the cell body as a reference point. The cell details were pseudocolored to highlight the dynamics of projections.
(0.94 MB AVI)

## Abbreviations

AP: anterior-posterior
AVE: anterior visceral endoderm
DVE: distal visceral endoderm
VE: visceral endoderm
