# Supplementary figures and images for: Rac1-Dependent Collective Cell Migration Is Required for Specification of the Anterior-Posterior Body Axis of the Mouse
Source: PLoS Biol. 2010 Aug 3;8(8):e1000442. doi: 10.1371/journal.pbio.1000442 (PMC2914637; doi:10.1371/journal.pbio.1000442)

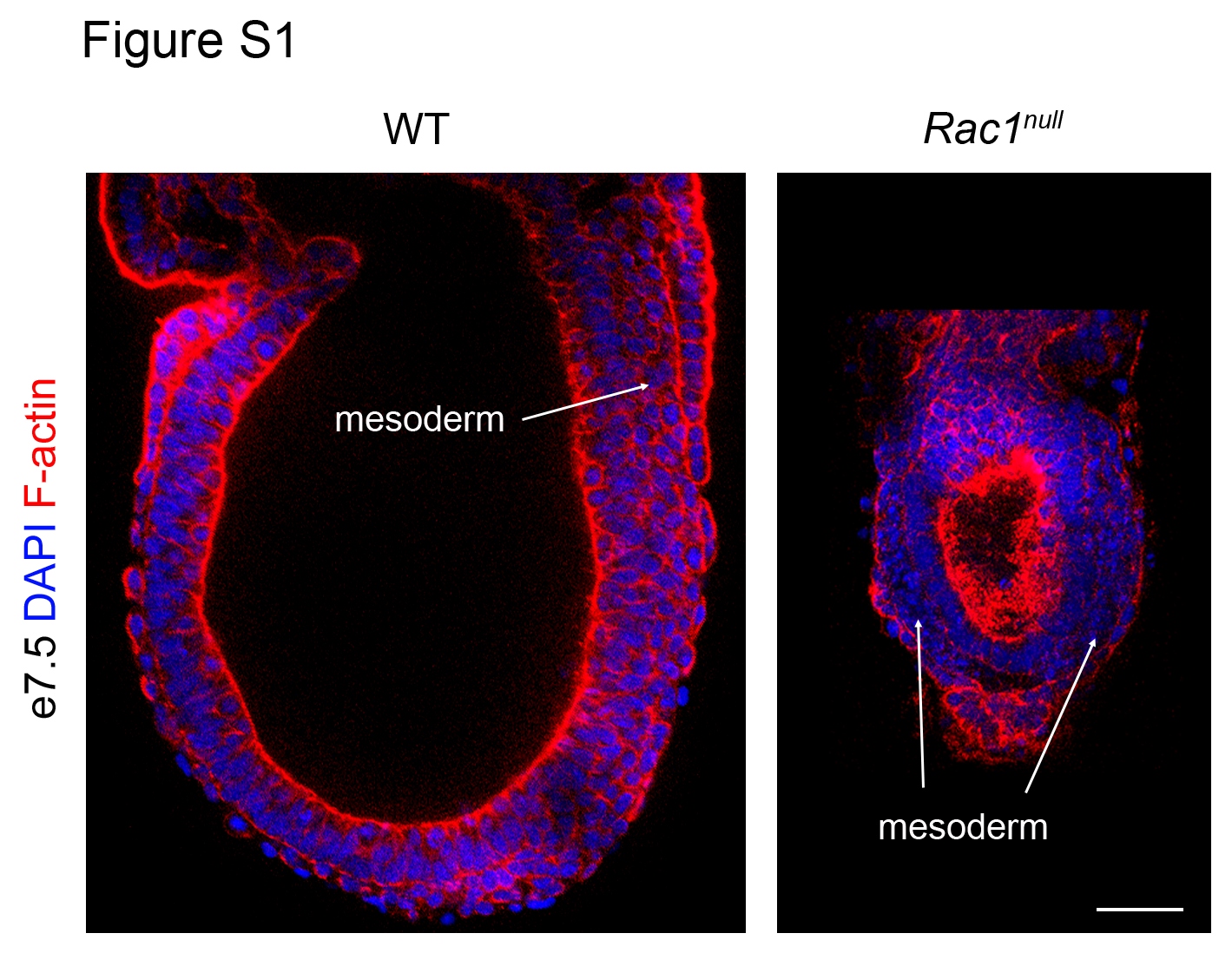

Supplement: Figure S1 — Gastrulation initiates in Rac1null embryos. Single confocal sections of e7.5 embryos stained with phalloidin to visualize F-actin (red). Mutant embryos generate mesoderm, which is present around the embryo between the epiblast and endoderm layers. There are numerous pyknotic nuclei in the mesoderm layer, as previously described [25]. Scale bars = 50 µm. (4.71 MB TIF) [file pbio.1000442.s001.tif]

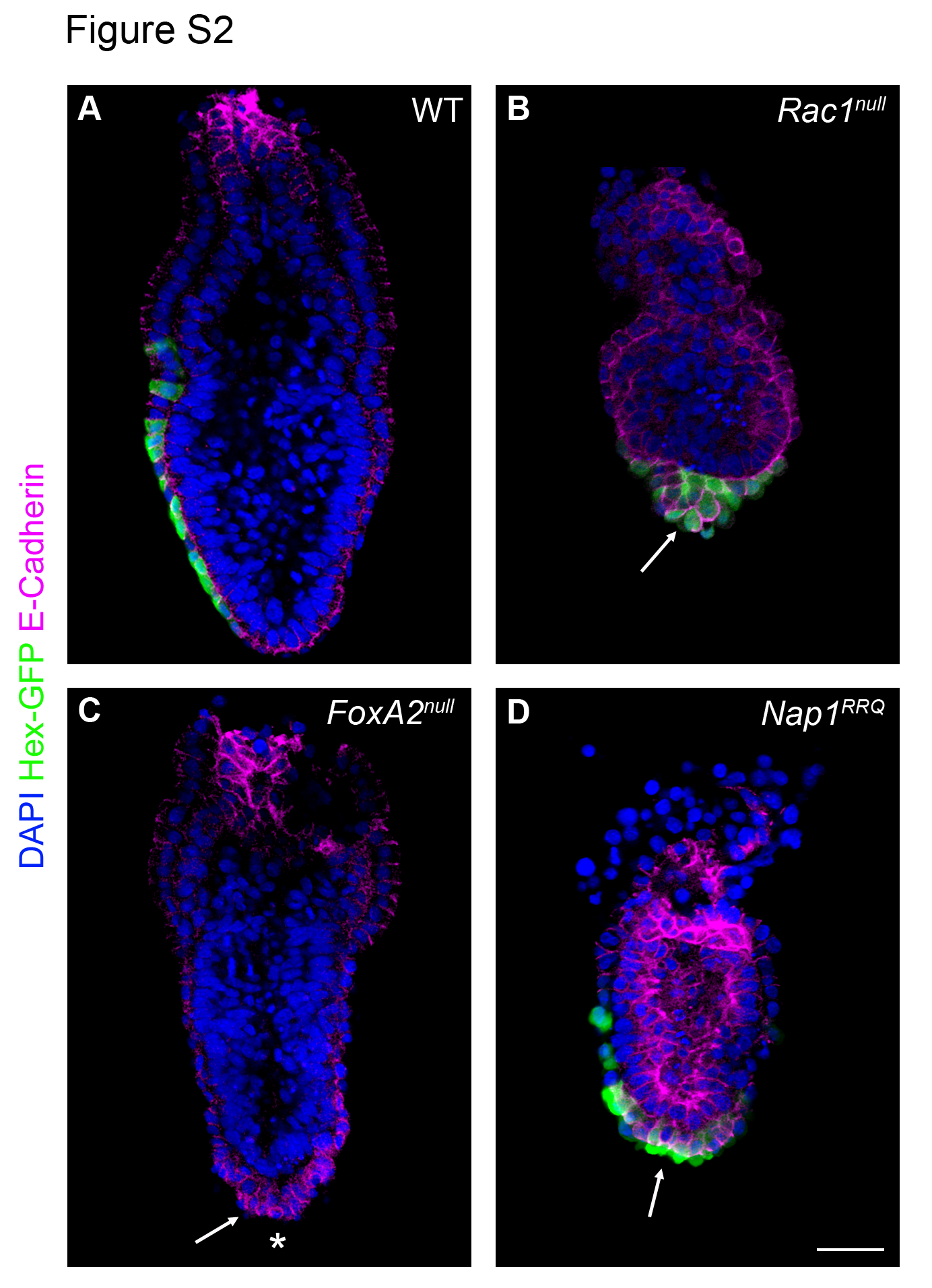

Supplement: Figure S2 — AVE cells that fail to migrate form a multilayered epithelium. Single confocal sections of e6.5 embryos stained for E-cadherin (magenta). At e6.5, AVE cells remained at the distal tip of the embryo in Rac1, FoxA2, and Nap1RRQ mutants expressing Hex-GFP (staining with anti-GFP antibody for wild-type, FoxA2, and RRQ embryos and native GFP for Rac1 null embryos). AVE cells formed several rows (arrows) and were linked by adherens junctions. In FoxA2 mutants (C), cells failed to express Hex-GFP but could be recognized through their columnar morphology (*). Nap1RRQ embryos (D) displayed a more severe phenotype than the Nap1khlo allele described in [10]. Scale bars = 50 µm. (7.97 MB TIF) [file pbio.1000442.s002.tif]

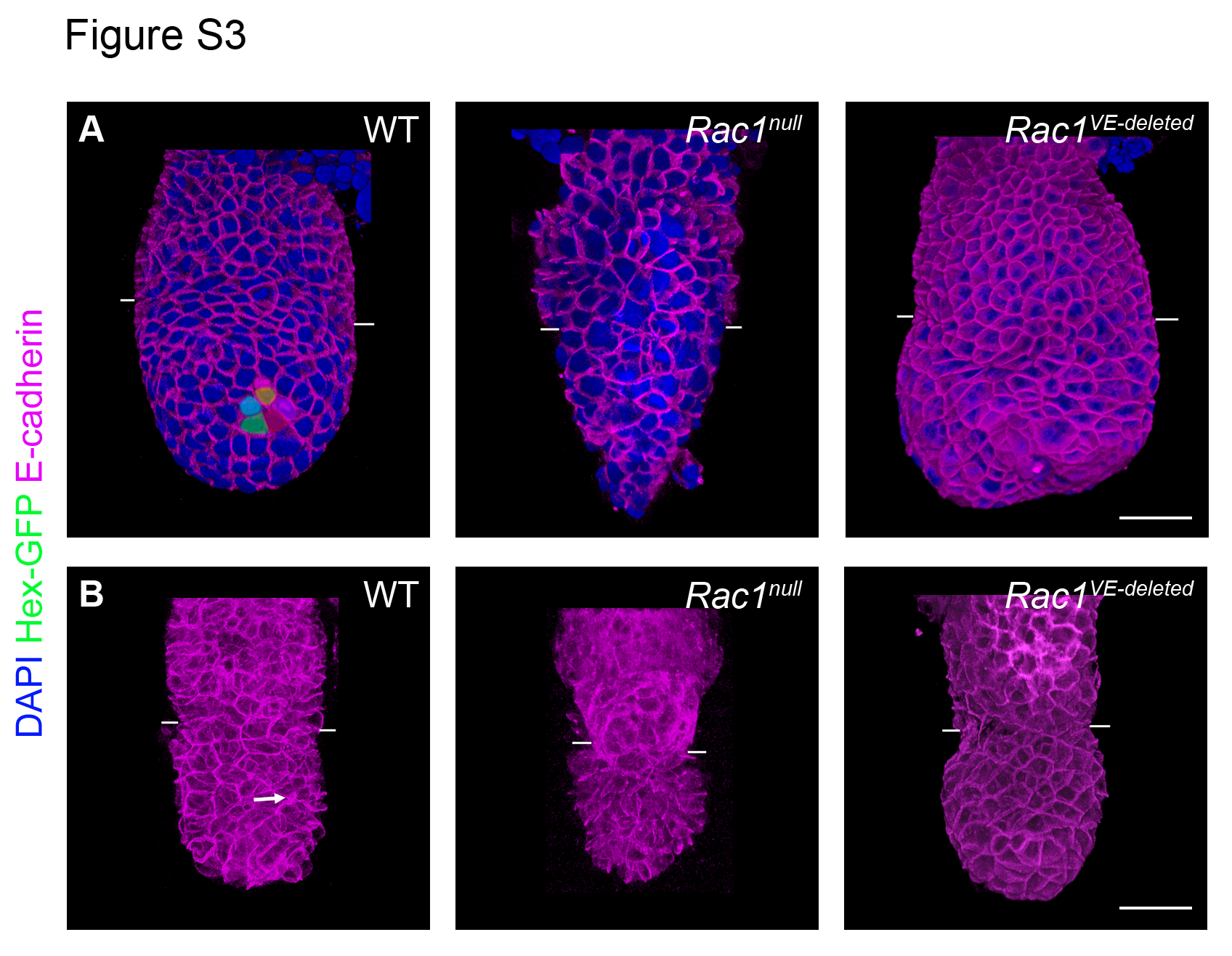

Supplement: Figure S3 — E-cadherin during migration. E-cadherin staining from the panels A and B of Figure 5. (A) Rosette-like structures can be detected in the wild-type VE (pseudo-colored). (B) The arrow points to the extremity of the long projection seen in Figure 5B. (6.73 MB TIF) [file pbio.1000442.s003.tif]

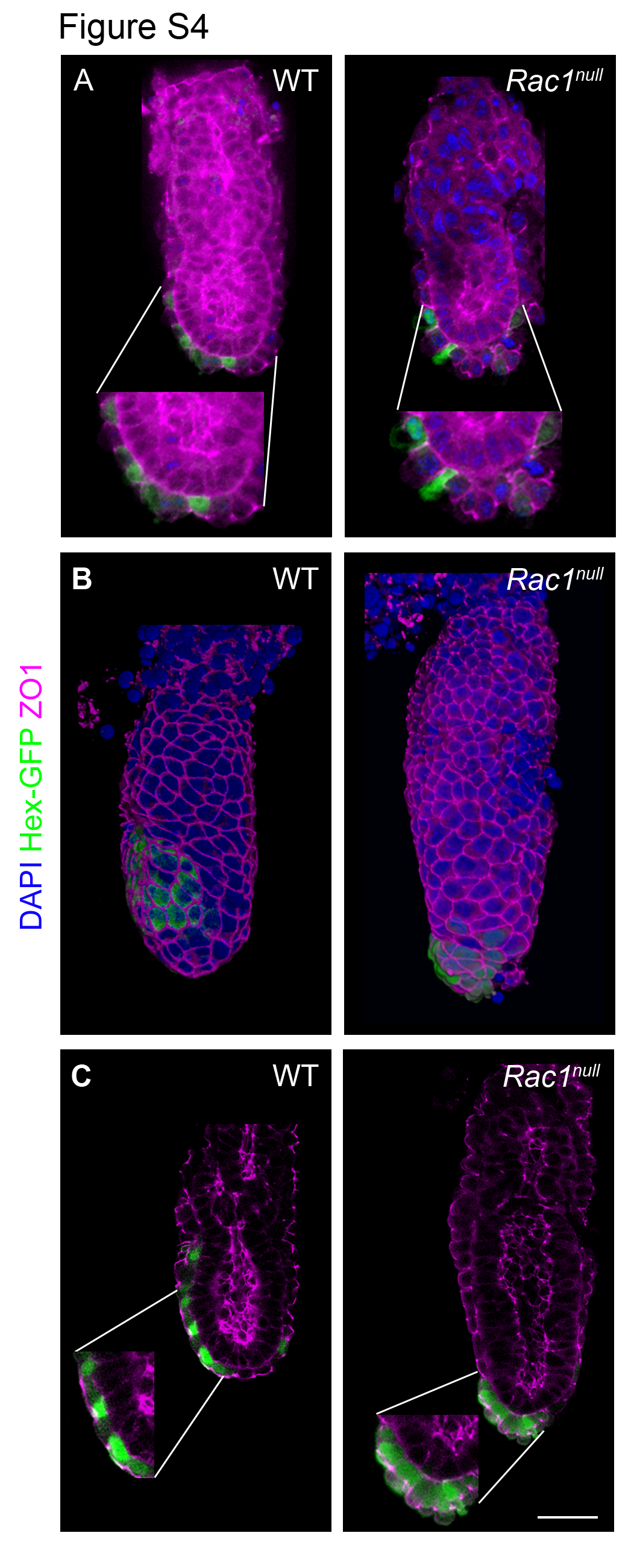

Supplement: Figure S4 — ZO1 during migration. (A) Individual confocal sections of e5.5–e5.75 embryos expressing Hex-GFP (green, detected with anti-GFP antibody) stained for ZO1 (magenta). In wild-type embryos, AVE cells retained apically localized tight junctions as they migrated. The VE of Rac1 null embryos showed a normal apical restriction of tight junctions at e5.5. However, between e5.5 and e5.75, some AVE cells lost contact with the basement membrane and expressed ZO1 on their lateral surfaces, prefiguring the cluster seen in Rac1 null embryos at e6.5 (see Figures 2 and S2). 3D reconstructions (B) and individual confocal sections (C) showing expression of Hex-GFP (green, native GFP) and ZO1 in stacks of e5.75 wild-type embryos and stage-matched Rac1 null embryos (dissected at e6.25). Tight junctions were normal in the VE of Rac1 null embryos at e6.25. Scale bars = 50 µm. Insets are 1.5×. (8.24 MB TIF) [file pbio.1000442.s004.tif]

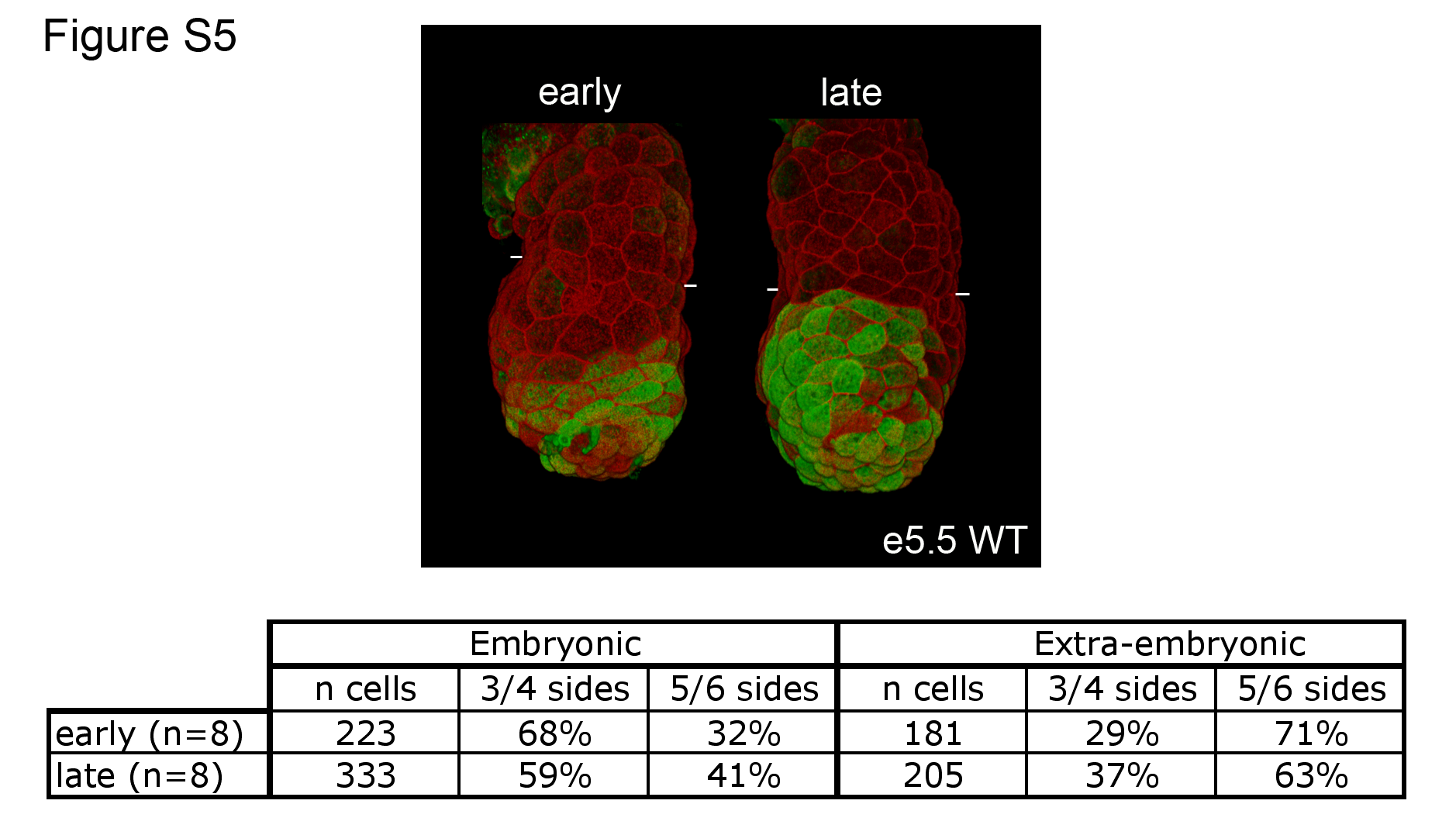

Supplement: Figure S5 — Cell shape is irregular in the embryonic VE during AVE migration. 3D reconstructions of Z-stacks of early (lateral view) and late (anterior view) embryos expressing Hex-GFP (green, native GFP), stained for F-actin (red). The embryos presented as examples were cultured for 1 h prior to fixation. A stable epithelium has a majority of pentagonal or hexagonal cells. The number of sides per cell in the extra-embryonic and embryonic regions of early and late e5.5 embryos was quantified on 3D reconstructions of Z-stacks, considering all faces of the embryos. All cells from the embryonic portion were considered, regardless of Hex-GFP expression, as all cells are likely to change shape either actively or passively. In both groups, most cells had 3 or 4 sides in the embryonic region, and 5 or 6 sides in the extra-embryonic region, and this trend was stronger in younger embryos in which the epithelium is expected to be less stable. (6.10 MB TIF) [file pbio.1000442.s005.tif]

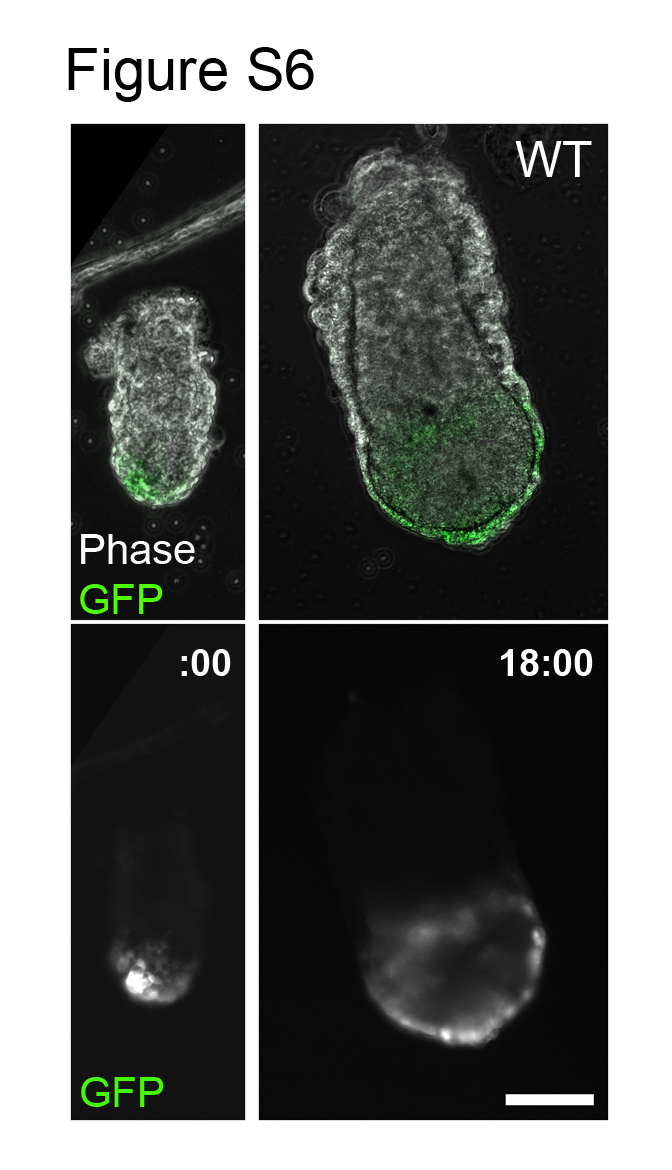

Supplement: Figure S6 — Embryo survival and growth in culture. Embryos dissected at e5.5 and cultured in a chamber at 37°C and 5% CO2 grow at close to normal rates under the culture conditions. Epifluorescence and phase contrast images of embryos expressing Hex-GFP at dissection and after 18 h of culture (used in Video S3). Scale bar = 100 µm. (2.39 MB TIF) [file pbio.1000442.s006.tif]

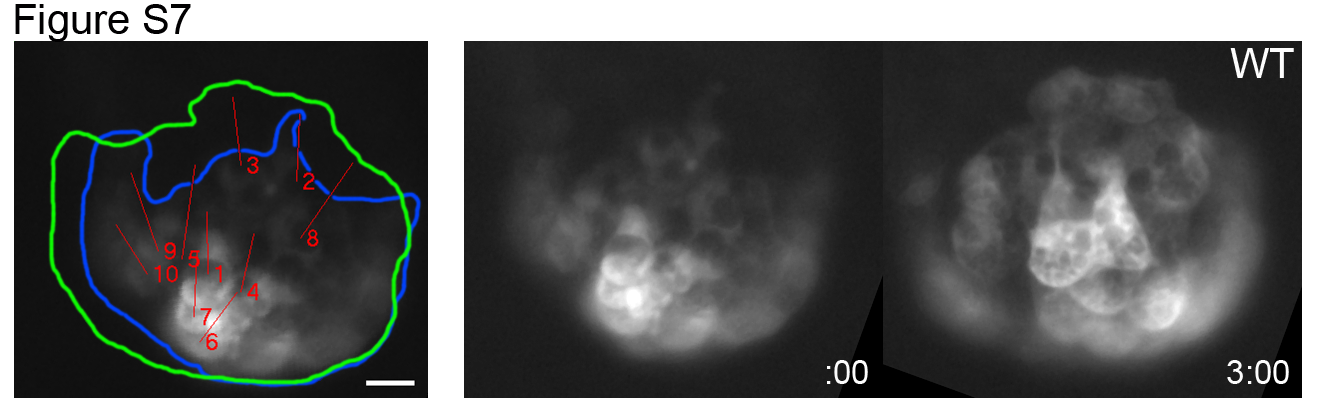

Supplement: Figure S7 — Vectors of migration of AVE cells. Epifluorescent time-lapse images (stills from Video S3) were aligned relative to the distal border of the embryo (at the bottom in Figure). In the left panel, the lines represent the initial (blue line, middle panel) and final (green line, right panel) outlines of Hex-GFP-expressing AVE cells population. The positions of individual cells were tracked and the cumulative vectors of migration were built using the “Track Points” function of Metamorph (numbered red lines). The average migration rate was 0.12±0.01 µm/min. The vector angle (assessing the directionality of migration) was 164±4.5 degrees relative to the bottom of images. Scale bar = 30 µm. (1.62 MB TIF) [file pbio.1000442.s007.tif]

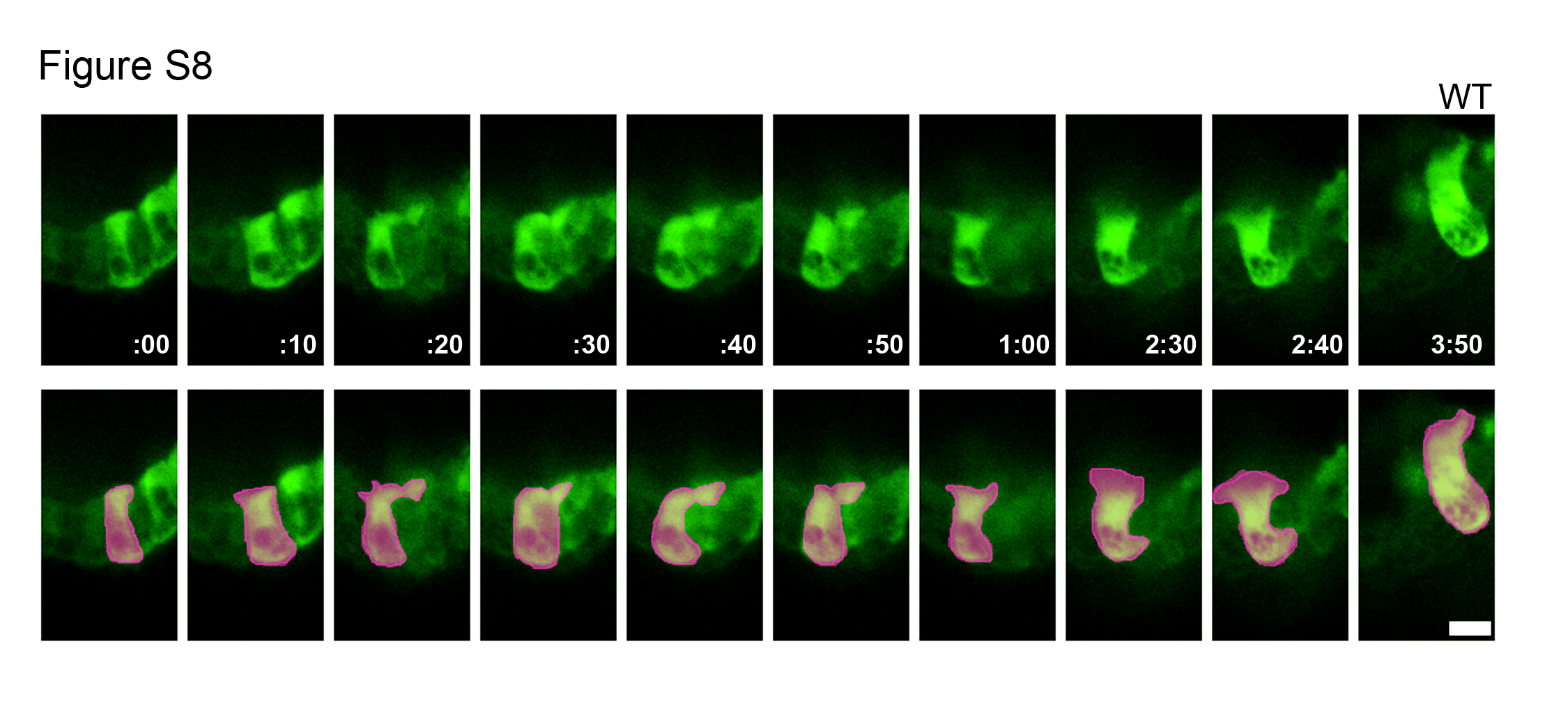

Supplement: Figure S8 — Basal projection of migrating AVE cell. Individual confocal images (stills from Video S5) of a trailing wild-type Hex-GFP cell. The cell showed basal protrusive activity while maintaining its columnar structure. The protrusion was directed towards the embryonic/extra-embryonic border (top right) and the cell translocated over time. Scale bar = 30 µm. (6.88 MB TIF) [file pbio.1000442.s008.tif]

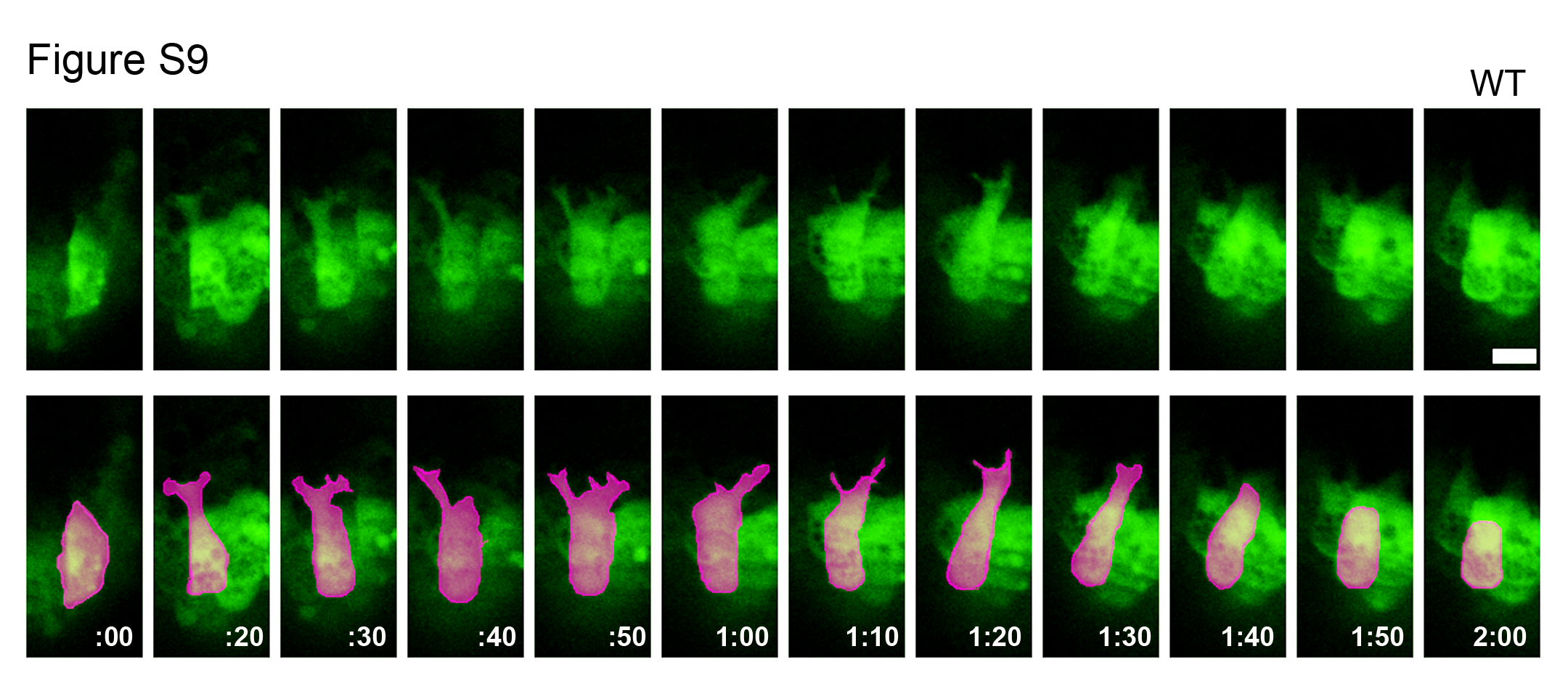

Supplement: Figure S9 — Front cell projection dynamics. Individual confocal sections (stills from Video S7) were aligned relative to center of the cell body and painted to highlight cell shape. The wild-type cell displayed is the blue cell in Figure 7B. The protrusive activity persisted for about 1.5 h. Protrusions had a stable direction for about 20 min (20–40 min), then retracted partially and extended at a slightly different angle (1 h 10 min–1 h 30 min). Scale bar = 30 µm. (6.16 MB TIF) [file pbio.1000442.s009.tif]

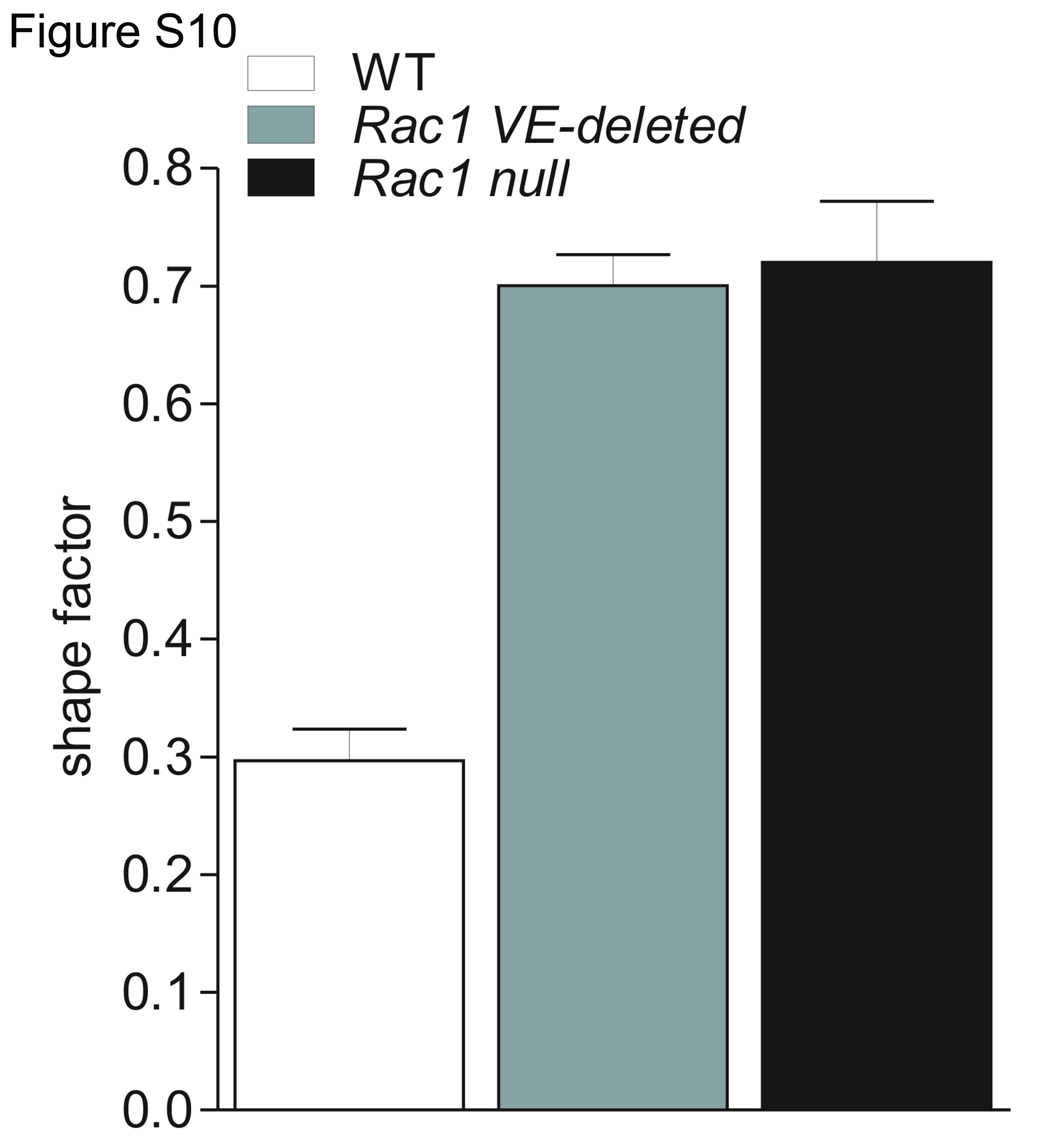

Supplement: Figure S10 — Rac1 mutant cells fail to elongate. The shape factor (see Methods) was calculated for cells from wild-type (12 cells from 8 embryos), Rac1 null (4 cells from 2 embryos), and Rac1 VE-deleted (9 cells from 5 embryos) from high resolution Z-stacks of live embryos. A value of 1 denotes a perfect circle, and lower values represent progressively more elongated or irregular shapes. Rac1 mutants are significantly rounder than wild type. Data are presented as mean ± SEM. (1.19 MB TIF) [file pbio.1000442.s010.tif]

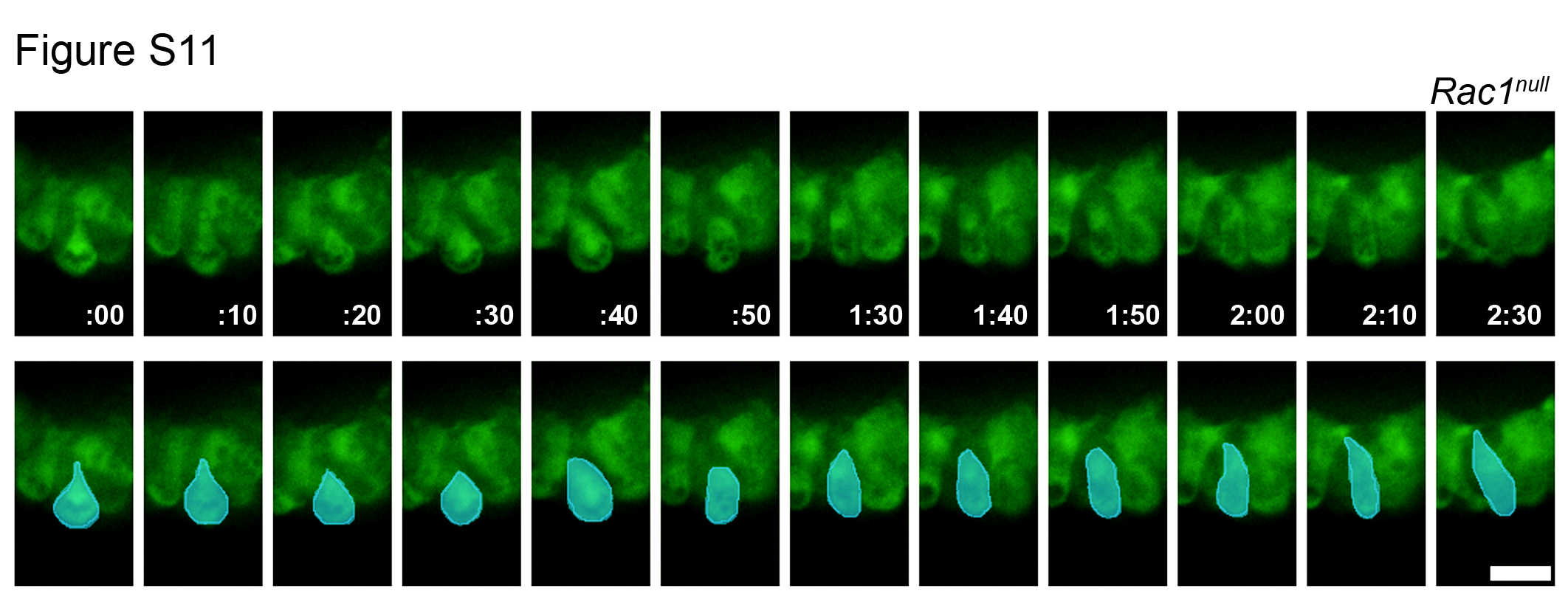

Supplement: Figure S11 — Formation of the distal cluster of DVE cells in a Rac1 null embryo. E6.0 Rac1 null mutant AVE cells were teardrop shaped and failed to make protrusions (stills from Video S10), and some cells appear to have lost contact with the basement membrane, which prefigures the formation of a grape-like cluster of AVE cells at the tip of the embryo. Scale bar = 30 µm. (5.28 MB TIF) [file pbio.1000442.s011.tif]

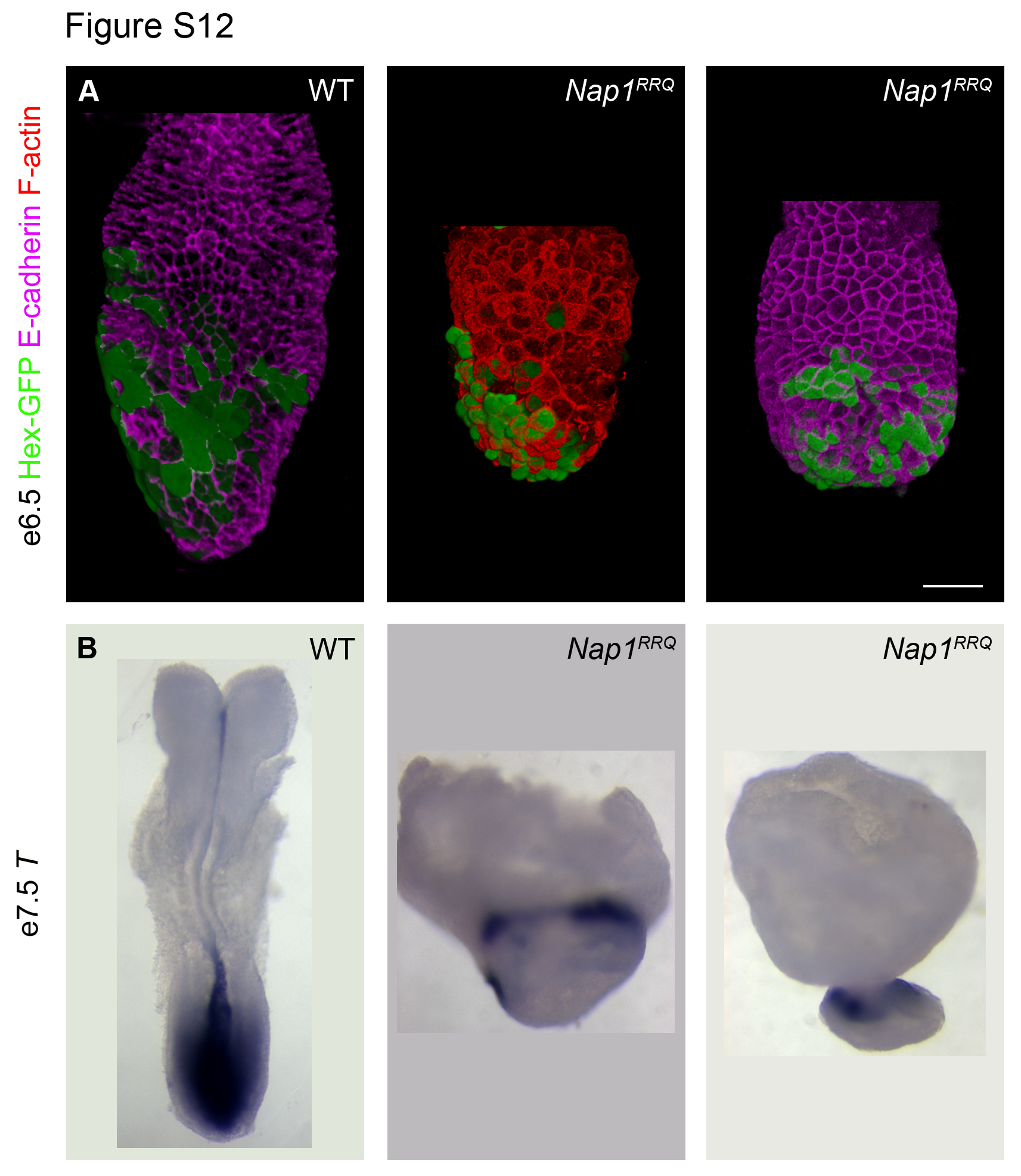

Supplement: Figure S12 — Defects in AVE migration and axis specification in Nap1RRQ embryos. (A) 3D reconstructions of Z-stacks of e6.5 embryos expressing Hex-GFP (green, staining with anti-GFP antibody), stained for F-actin (red) or E-cadherin (magenta). In Nap1RRQ embryos, the distribution of Hex-GFP is abnormal, and many cells fail to initiate migration. Cells are round in the VE of mutant embryos, reminiscent of the Rac1 mutant embryos. (B) Expression of Brachyury (T) at e7.5. In Nap1RRQ mutants, there appear to be multiple sites of streak initiation or, in the most severe cases, a ring of T-expressing cells in the proximal epiblast. (10.18 MB TIF) [file pbio.1000442.s012.tif]
